# Microwave-induced degradation of Congo red dye in the presence of 2D Ti_3_C_2_T_x_ MXene as a catalyst

**DOI:** 10.1038/s41598-024-82911-9

**Published:** 2025-01-03

**Authors:** Salma M. El-Mas, Mohamed A. Hassaan, Gehan M. El-Subruiti, Abdelazeem S. Eltaweil, Ahmed El Nemr

**Affiliations:** 1 Department of Chemistry, Faculty of Science, Alexandria University, Alexandria, Egypt; 2 Environment Division, National Institute of Oceanography and Fisheries (NIOF), Kayet Bey, Elanfoushy, Alexandria Egypt

**Keywords:** Congo red dye, Microwave-induced degradation, 2D Ti_3_C_2_T_x_ MXene, RSM optimization, Scavengers impact, Environmental chemistry, Environmental chemistry, Pollution remediation

## Abstract

In this research, the degradation of Congo red (CR) dye, as an organic pollutant in water, was investigated using microwave-induced reaction technology. This technology requires a microwave-absorbing catalyst and the 2D Ti_3_C_2_T_x_ MXene was synthesized for that purpose. The synthesized catalyst was characterized using XRD, SEM, TEM, EDX, BET, and XPS techniques. Results showed that the prepared 2D Ti_3_C_2_T_x_ MXene with a dosage of 50 mg degraded CR dye with an initial concentration of 25 ppm in an aqueous solution with a degradation percentage of approximately 99% in only 6 min. The parameters studied were catalyst dosage and initial CR dye concentration, which were found to have significant impacts on the degradation rate. When the catalyst dosage was increased significantly, the degradation rate increased significantly. On the other hand, when increasing the initial CR dye concentration, the degradation rate decreased. The degradation kinetics were studied, and the reaction followed the pseudo-first-order model. The rate constants obtained ranged from 0.04 to 0.83 min^−1^, varying according to the used catalyst dosage and initial CR dye concentration. The catalyst was stable and could be reused for up to five catalytic cycles without losing its degradation efficiency. The active species participating in the degradation process were determined using scavengers such as benzoquinone, Na-EDTA, and isopropyl alcohol. Optimization of the degradation parameters using a response surface methodology study concluded that a maximum degradation percentage could be reached when employing 35.30 mg of 2D Ti_3_C_2_T_x_ MXene and 29.07 ppm of CR dye solution.

**Supplementary Information:**

The online version contains supplementary material available at 10.1038/s41598-024-82911-9.

## Introduction

Due to severe industrial water pollution and an increasing population, access to clean water is becoming a rising challenge. Water bodies are being exposed to a large number of organic pollutants from industries including textile, paint, and leather. These organic contaminants, which are extremely difficult to remove by conventional wastewater treatment methods, have significant negative impacts on aquatic systems and human health^1–4^. Considering organic dyes as an example of these organic pollutants, there are more than 100,000 commercially available dyes with more than 7 × 10^5^ tons manufactured per year. These dyes pose a significant risk to the environment since they are chemically stable and do not degrade in water. Solutions to treat these dyes with high efficiency and low cost are therefore urgently needed, given the growing focus on human health and environmental protection^5–7^.

Congo red (CR) dye is one of the toxic anionic azo dyes. Due to its affinity for cellulose fibres, it finds extensive use in the textile industry. It is used to diagnose amyloidosis and as a pH indicator. However, CR dye has several negative impacts. It could be converted into benzidine, a human carcinogen. The dye is mutagenic and causes allergic responses. It can lead to gastrointestinal problems and respiratory illnesses. Its complex structure gives it a remarkable degree of stability and resistance to degradation^7,8^.

Many researches^9–12^ focused on adsorption methods to remove organic pollutants from wastewater, considering their simple design and easy operation. However, they only transfer pollutants from one phase to another and reduce their concentration rather than removing them^5^.

In contrast, microwave-induced reaction technology could completely mineralize organic pollutants into non-toxic inorganics CO_2_ and H_2_O under mild conditions without secondary pollution. This technology has been gaining more and more attention lately. This method can effectively decompose organic contaminants and has the potential to save energy and shorten reaction times. It is generally known that using the suitable microwave-absorbing materials in a microwave-induced reaction system can speed up the reaction time and accelerate the breakdown of the target compounds. Therefore, when using a microwave to generate a reaction, it is crucial to select a suitable microwave catalyst^13^. Microwave absorbents, including metallic particles^14–16^, activated carbon^17^, and polymers^18^, have recently received a lot of attention in the wastewater treatment industry. In microwave catalytic degradation of organic molecules, these materials are typically employed.

Mxenes, a novel family of 2D transition metal carbides, nitrides, and carbonitrides, have attracted significant attention in the field of microwave absorption. The general chemical formula for MXenes is M_*n*+1_X_n_T_x_, where the transition metal is denoted by M, C, and/or N is represented by X, and surface termination (-O, -OH, or -F) is denoted by T_x_^19–21^. They are typically synthesized by selectively etching the A layer in a solution containing hydrofluoric acid from their precursor MAX phases (M_*n*+1_AX_n_, where A is an element from groups 13 or 14)^22,23^. MXenes exhibit several remarkable chemical and physical characteristics, including high specific surface area, surface hydrophilicity, excellent conductivity, and a chemically active surface, which make MXenes candidates for many applications in the field of microwave absorption^24–27^. Among the most well-known MXene compounds is Ti_3_C_2_T_x_, which has attracted considerable attention in microwave absorption^28–30^.

Although Ti_3_C_2_T_x_ MXene has been extensively used, there have been no studies made on the use of Ti_3_C_2_T_x_ as a microwave-absorbing catalyst in the degradation of CR dye. In this study, 2D Ti_3_C_2_T_x_ MXene was synthesized using the typical hydrofluoric-etching method, and characterization techniques, including XRD, SEM, TEM, EDX, BET and XPS, were employed to study the structural and chemical characteristics of the synthesized 2D Ti_3_C_2_T_x_ MXene. The degradation of CR dye under microwave irradiation in the presence of 2D Ti_3_C_2_T_x_ MXene as a microwave absorber was investigated. Moreover, the impact of different parameters, including catalyst dosage and initial CR dye concentration, on the degradation rate was determined. In addition, the main active species responsible for the degradation of CR dye was investigated using a scavenger test. Also, response surface methodology (RSM) optimization of CR dye degradation in water was conducted.

## Experimental

### Materials

All the chemical reagents used are of analytical grade and are used without further purification. Titanium aluminium carbide Ti_3_AlC_2_ powder (≥ 90%, ≤ 40 μm particle size) was obtained from Sigma-Aldrich. Hydrofluoric acid HF solution (40%) was purchased from ISOLAB Chemicals, India, and the targeted pollutant (CR dye) (Fig. S1) was purchased from Alfa Aesar, India. Deionized water was utilized in all experiments.

### Preparation of Ti3C2Tx MXene nanosheets

The 2D Ti_3_C_2_T_x_ MXene was prepared using the typical hydrofluoric-etching method in which the precursor Ti_3_AlC_2_ MAX phase (5 g) was slowly added to a solution of hydrofluoric acid 40% (150 mL) in a TEFLON autoclave. The mixture was then kept on a magnetic stirrer to react for approximately 24 h. After that, the resulting suspension was washed carefully and thoroughly with deionized water many times until reaching pH ≥ 6. Lastly, an oven adjusted at 50 °C was used to dry the obtained 2D Ti_3_C_2_T_x_ MXene nanosheets overnight.

### Characterization

A scanning electron microscope (SEM) equipped with an energy dispersive x-ray (EDX) instrument (JEOL JSM-IT200 Series, Japan) was utilized to examine the elemental composition and surface morphology. A Transmission Electron Microscope (TEM, JEOL JEM-1400Plus, Japan) was employed to evaluate the distribution of particle sizes. The crystal structure and crystallinity of the precursor Ti_3_AlC_2_ MAX phase and synthesized 2D Ti_3_C_2_T_x_ MXene were explored using a Bruker Meas Srv X-ray diffractometer (XRD, D2-diffractometer) that operates at 30 kV and 10 mA using Cu-Kα radiation source of *λ* = 1.5418 Å and 2*θ* with a temperature range of 5 to 80 °C. The Brunauer-Emmett-Teller (BET, BELSORP Mini II, BEL Japan, Inc) method was used to measure the specific surface area and mean pore diameter of the obtained material. The elemental chemical states and composition were determined using X-ray photoelectron spectroscopy (XPS, ESCALAB 250Xi, ThermoFisher Scientific, USA) using Al-Kα radiation (*hν* = − 10 to 1350 eV). The characterization of the Ti_3_AlC_2_ MAX phase and synthesized 2D Ti_3_C_2_T_x_ MXene were explained in detail in the supporting materials (Figures S2-S6 and Tables S1 and S2).

### Microwave degradation of CR

The microwave catalytic performance of the synthesized 2D Ti_3_C_2_T_x_ MXene was tested by the decomposition of CR dye. Typically, in a glass reactor, a certain amount of 2D Ti_3_C_2_T_x_ MXene powder was added to 50 mL of CR dye aqueous solution at a pH of 6, without adding HCl or NaOH. Afterward, the reactor was placed in a domestic microwave oven and connected to a condenser to prevent volatilization of the solvent. Then, the suspension was irradiated by microwave (700 W) for a measured amount of time. At certain time intervals, a sample was taken out and left to cool down. After that, it was centrifuged and the supernatant liquid was analysed by a UV-Vis spectrophotometer at *λ*_max_ 498 nm, the maximum absorption wavelength of CR dye.

Moreover, the impact of initial pH on the degradation rate was studied by carrying out the reaction at different pH values ranging from 2 to 10. Catalyst dosage, reaction time, and initial concentration were in ranges of 30–50 mg, 0–16 min, and 25–125 mg/L, respectively. The degradation percentage was calculated by the following Eq. (1)^31–33^:

1 $$\:\text{Degradation}\:\%=\left(\frac{{\text{C}}_{0}-\text{C}}{{\text{C}}_{0}}\:\right)\times\:100\%=\left(\frac{{\text{A}}_{0}-\text{A}}{{\text{A}}_{0}}\right)\times\:100\%$$

where *C*_0_ and *A*_0_ represent initial concentration and absorbance, and *C* and *A* represent concentration and absorbance at a particular time. The degradation of CR dye was found to fit the pseudo-first-order kinetic model as Eq. (2)^31,34^:

2 $$\:\text{ln}\left(\frac{{\text{C}}_{0}}{\text{C}}\right)=\text{kt}$$

where *C* is the CR dye concentration at reaction time *t*, *C*_0_ is the initial concentration of CR dye before the reaction and k is the reaction decomposition rate constant.

The dye adsorption with the 2D Ti_3_C_2_T_x_ MXene was performed and left for 30 min in the dark to attain adsorption-desorption equilibrium, and only ~ 1% of CR dye was removed. Moreover, the degradation without adding a catalyst to test the effect of microwave irradiation alone was also performed. In the absence of 2D Ti_3_C_2_T_x_ MXene, only ~ 1.5% of CR dye was degraded under MW irradiations for 50 ppm initial CR dye concentration.

In addition, the effect of the precursor Ti_3_AlC_2_ MAX phase on the reaction rate was detected. Furthermore, the recyclability of the synthesized 2D Ti_3_C_2_T_x_ MXene catalyst was tested. Finally, to further understand the degradation mechanism and determine the main active species responsible for the degradation of CR dye, scavengers, including ethylenediaminetetraacetic acid disodium salt (Na-EDTA), isopropyl alcohol (IPA) and *p*-benzoquinone (BQ), were employed.

### RSM

Optimization of parameters affecting CR dye degradation in the presence of 2D Ti_3_C_2_Tx MXene as a catalyst under MW irradiation was studied using response surface methodology (RSM). The software employed was Design-Expert version 13.0.5.0, and the Box-Behnken design (BBD) was utilized. Reaction time, catalyst dosage, and initial CR dye concentration were selected for this purpose^35–41^. The studied parameters and corresponding levels are reported in Table 1. The response studied was CR dye removal percent (%). A total of 17 experiments with different combinations of variables were conducted. The designed experiments are displayed in Table 1.

**Table 1:** The range of studied parameters used in the optimization process.

Factor	Name	Units	Minimum	Maximum	Mean	Std. dev.
A	Dose	Mg	30.00	50.00	30.00	7.07
B	Conc.	mg/L	25.00	125.00	75.00	35.36
C	Time	Min	4.00	12.00	8.00	2.83

## Results and discussion

### Degradation of CR

#### Microwave degradation test

The catalytic ability of the precursor Ti_3_AlC_2_ MAX phase and the synthesized 2D Ti_3_C_2_T_x_ MXene was investigated. Also, the degradation reaction of CR dye was carried out under microwave irradiation without adding a catalyst. In the case of adding Ti_3_AlC_2_ MAX to the reaction mixture, the amount of CR dye molecules degraded was negligible (~ 1.3%), meaning that Ti_3_AlC_2_ MAX is not an effective microwave catalyst in CR dye degradation. Furthermore, without adding a catalyst to the reaction medium, no CR dye was degraded. On the other hand, in the presence of the synthesized 2D Ti_3_C_2_T_x_ MXene, the degradation efficiency could reach, in some cases, approximately 99% in a short time. This means that almost all CR dye molecules could be degraded and complete degradation could be achieved, indicating that 2D Ti_3_C_2_T_x_ MXene is an effective and highly efficient microwave-absorbing catalyst for CR dye degradation.

#### Effect of initial pH

**Fig. 1:**
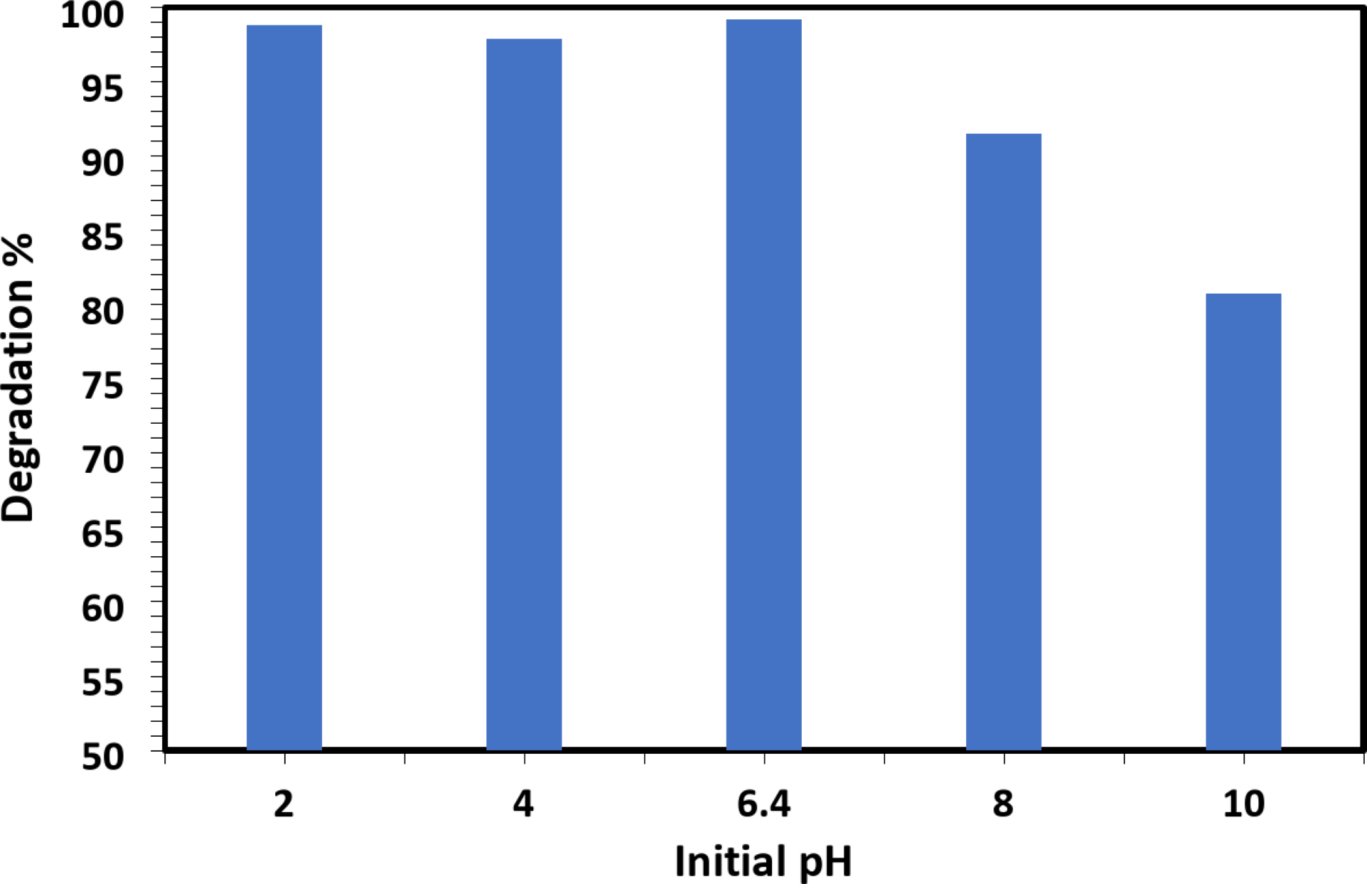
Variation of degradation efficiency of CR dye with initial pH 2–10 using 2D Ti_3_C_2_T_x_ Mxene as catalyst for 16 min.

The degradation reaction was carried out at different initial pH values ranging from 2 to 10 to study the effect of pH on CR dye degradation rate. At all studied pH values (pH = 2, 4, 6.4, 8, and 10), the CR dye degradation reaction took place with high degradation efficiencies, as shown in Fig. 1. At pH = 10, the lowest degradation efficiency (80.77%) was obtained, while at the natural pH of CR dye solution (pH = 6.4), the highest degradation efficiency (99.23%) was achieved. As a result, all experiments were conducted at the natural pH of CR dye solution without adjusting the pH (without adding HCl or NaOH).

2D Ti_3_C_2_T_x_ MXene surfaces typically have a small positive or neutral charge at pH 6.4 (neutral to slightly acidic), which might encourage the adsorption of negatively charged dyes (such as anionic dyes). This improves removal efficiency by strengthening the electrostatic interaction between the CR dye and the 2D Ti_3_C_2_T_x_ MXene surface. On the other hand, the deprotonation of functional groups (such as hydroxyls) causes the 2D Ti_3_C_2_T_x_ MXene surface to become more negatively charged at pH 10. Electrostatic repulsion happens if the dye is also negatively charged, which lowers the adsorption effectiveness and, consequently, the amount of dye removed. Furthermore, pH may have an impact on the production of reactive species (such as hydroxyl radicals ^•^OH) during catalytic dye degradation. The environment is more favourable for the production of these radicals at pH 6.4, which speeds up the dye breakdown process. At pH 10, on the other hand, radical generation efficiency may drop or produced radicals may quickly recombine, decreasing their availability for dye degradation. The effectiveness of dye removal at higher pH values is constrained by this phenomenon.

#### Effect of catalyst dosage and initial CR concentration

**Fig. 2:**
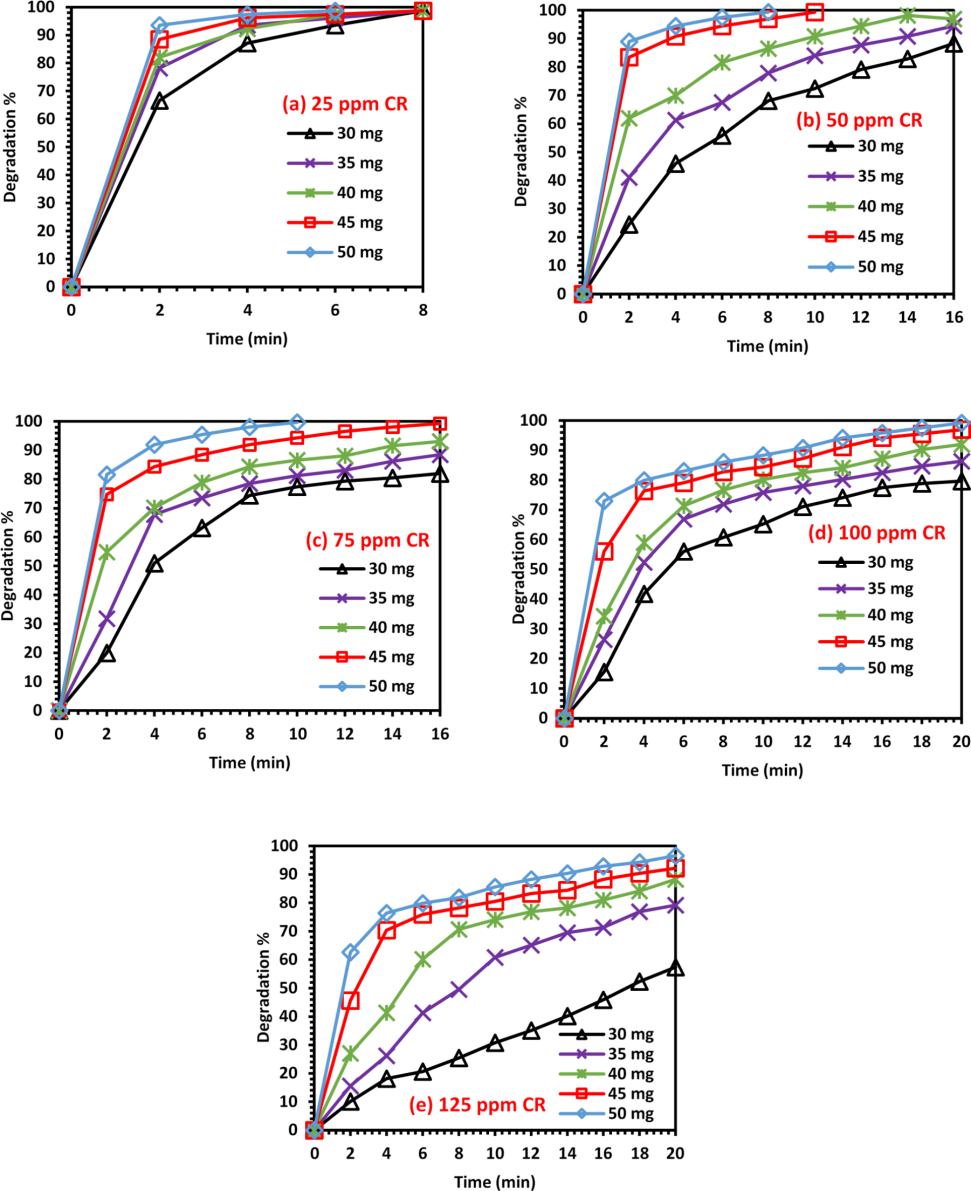
The effect of initial CR dye concentration on its degradation: (**a**) 25, (**b**) 50, (**c**) 75, (**d**) 100 and (**e**) 125 ppm.

**Fig. 3:**
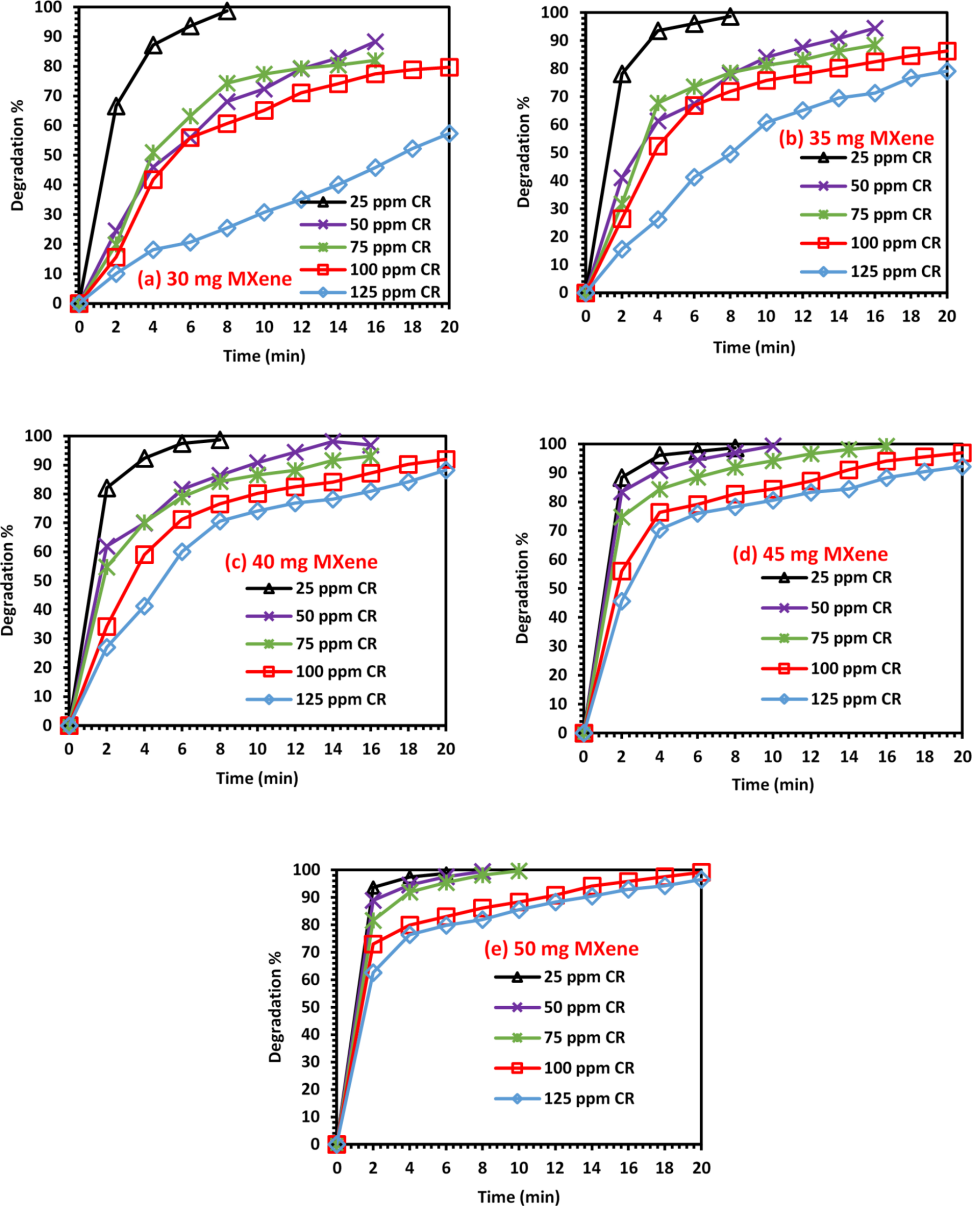
The effect of catalyst dosage on CR dye degradation: (**a**) 30, (**b**) 35, (**c**) 40, (**d**) 45, and (**e**) 50 mg of the 2D Ti_3_C_2_T_x_ Mxene catalyst.

To study the effect of catalyst dosage and initial CR dye concentration on the degradation rate, the reaction was carried out using different amounts of 2D Ti_3_C_2_T_x_ Mxene (30, 35, 40, 45, and 50 mg) and different initial CR dye concentrations (25, 50, 75, 100 and 125 ppm). Figures 2 and 3 show that both catalyst dosages and initial CR dye concentrations significantly affect the CR dye degradation. For each initial CR dye concentration, the reaction was carried out using five different catalyst dosages. It could be noticed that at the same initial CR dye concentration, the degradation rate and efficiency increased when the catalyst dosage was increased. This could be attributed to the availability of more active sites for the same amount of CR dye molecules. In addition, for each catalyst dosage, five initial CR dye concentrations were examined. It was found that the reaction rate and degradation efficiency decreased for the same catalyst dosage when using a higher initial CR dye concentration^42–46^. This is because more CR dye molecules need to be degraded using a constant number of active sites, decreasing the rate of degradation. The highest degradation efficiencies obtained when using initial CR dye concentrations of 25, 50, 75, 100, and 125 ppm were 98.7, 99.4, 99.6, 99.2 and 96.6%, respectively. This was achieved using the highest catalyst dosage of 50 mg after 6, 8, 10, 20 and 20 min, respectively. Moreover, the highest degradation efficiency obtained when using catalyst dosages 30, 35, 40, 45 and 50 mg was nearly 99% in 6–8 min when using the lowest initial CR dye concentration (25 ppm). When employing the highest catalyst dosage (40 mg) and lowest initial CR dye concentration (25 ppm), a high degradation efficiency of 98.7% was achieved in only 6 min. It could be concluded that optimum degradation could be reached by increasing the dosage of 2D Ti_3_C_2_T_x_ MXene and decreasing the initial concentration of the dye used.

### Reaction kinetics

**Fig. 4:**
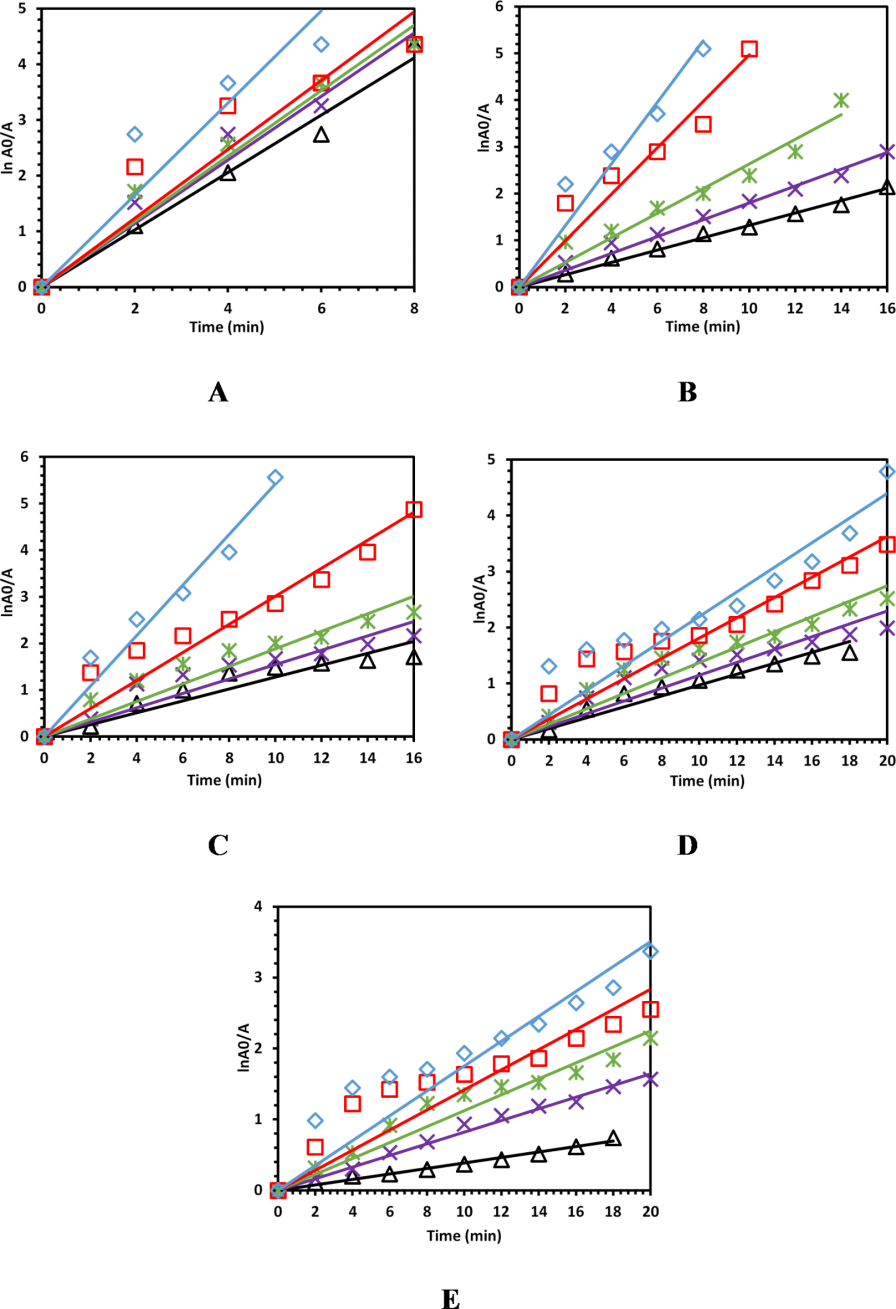
The effect of catalyst dosage on degradation rate using different initial concentrations (**A**) 25, (**B**) 50, (**C**) 75, (**D**) 100, and (**E**) 125 ppm of CR dye.

The reaction kinetics of the microwave-induced degradation of CR dye was investigated. Figure 4 displays the kinetic plots of CR dye degradation using different catalyst dosages and initial CR dye concentrations. The high values of linear correlation coefficients (*R*^2^) shown in Table 2 confirmed that CR dye degradation obeys the pseudo-first-order kinetic model. By observing the values of *k* displayed in Table 2, it could be concluded that catalyst dosages and initial CR dye concentrations had significant impacts on the CR dye degradation rate. Higher catalyst dosages enhanced the degradation of CR dye, and higher initial CR dye concentrations retarded the dye degradation. The lowest *k*-value (0.0396 min^−1^) was obtained at the lowest catalyst dosage (30 mg) and highest initial CR dye concentration (125 mg/L), while the highest *k*-value (0.8266 min^−1^) was reached when employing the highest catalyst dosage (50 mg) and smallest initial CR dye degradation (25 mg/L). Increasing the catalyst dosage will improve dye degradation as more surface area will be available for degradation, and hence, the number of active sites on the 2D Ti_3_C_2_T_x_ Mxene surface will increase. On the other hand, as for increasing the initial CR dye concentration, the degradation rate will decrease because more pollutant molecules are adsorbed onto the catalyst surface, which has a limited number of active sites^47–51^.

**Table 2:** Values of pseudo-first-order rate constants and *R*^2^ for CR dye microwave-induced degradation using different catalyst dosages and initial CR dye concentrations.

Initial dye concentration (ppm)	Catalyst dosage (mg)	Rate constant (min^−1^)	*R* ^2^
25	30	0.5146	0.9944
	35	0.5703	0.9890
	40	0.5878	0.9886
	45	0.6182	0.9619
	50	0.8266	0.9577
50	30	0.1321	0.9981
	35	0.1801	0.9958
	40	0.2033	0.9914
	45	0.4970	0.9807
	50	0.6582	0.9822
75	30	0.1277	0.9716
	35	0.1545	0.9664
	40	0.1883	0.9713
	45	0.3010	0.9826
	50	0.5420	0.9894
100	30	0.0928	0.9812
	35	0.1148	0.9708
	40	0.1373	0.9793
	45	0.1812	0.9783
	50	0.2196	0.9738
125	30	0.0396	0.9950
	35	0.0821	0.9969
	40	0.1124	0.9843
	45	0.1416	0.9620
	50	0.1753	0.9688

### The effect of scavengers on CR degradation

**Fig. 5:**
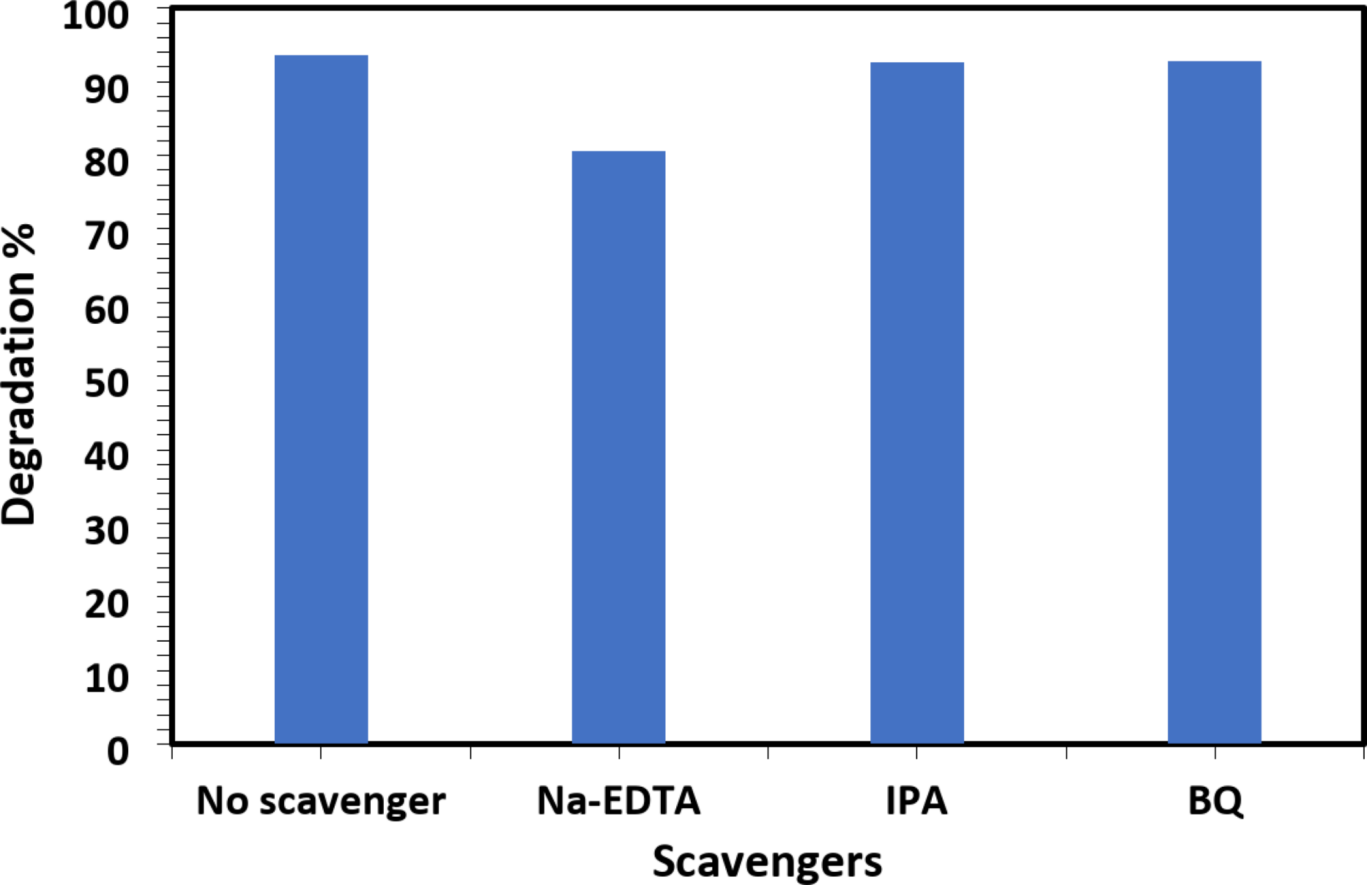
Effect of diverse scavengers on degradation % of CR dye.

To understand the mechanism of microwave-induced degradation of CR dye concentration of 75 ppm and catalyst dosage of 40 mg of 2D Ti_3_C_2_T_x_ MXene, scavengers, such as Na-EDTA 0.452 mmol, IPA 0.0653 mol, and BQ 0.0925 mmol, were added to the reaction medium. Na-EDTA was used as a hole (h^+^) scavenger, IPA was used as a hydroxyl radical (.OH) scavenger and BQ was employed as a superoxide radical (.O_2_^–^) scavenger. Results, as shown in Fig. 5, both IPA (%*R* = 92.56%) and BQ (%*R* = 92.83%) had no considerable effect on the CR dye degradation while the addition of Na-EDTA caused a noticeable decrease in the removal percentage of CR dye (%*R* = 80.64%), if compared to that without adding a scavenger (%*R* = 93.55%).

**Fig. 6:**
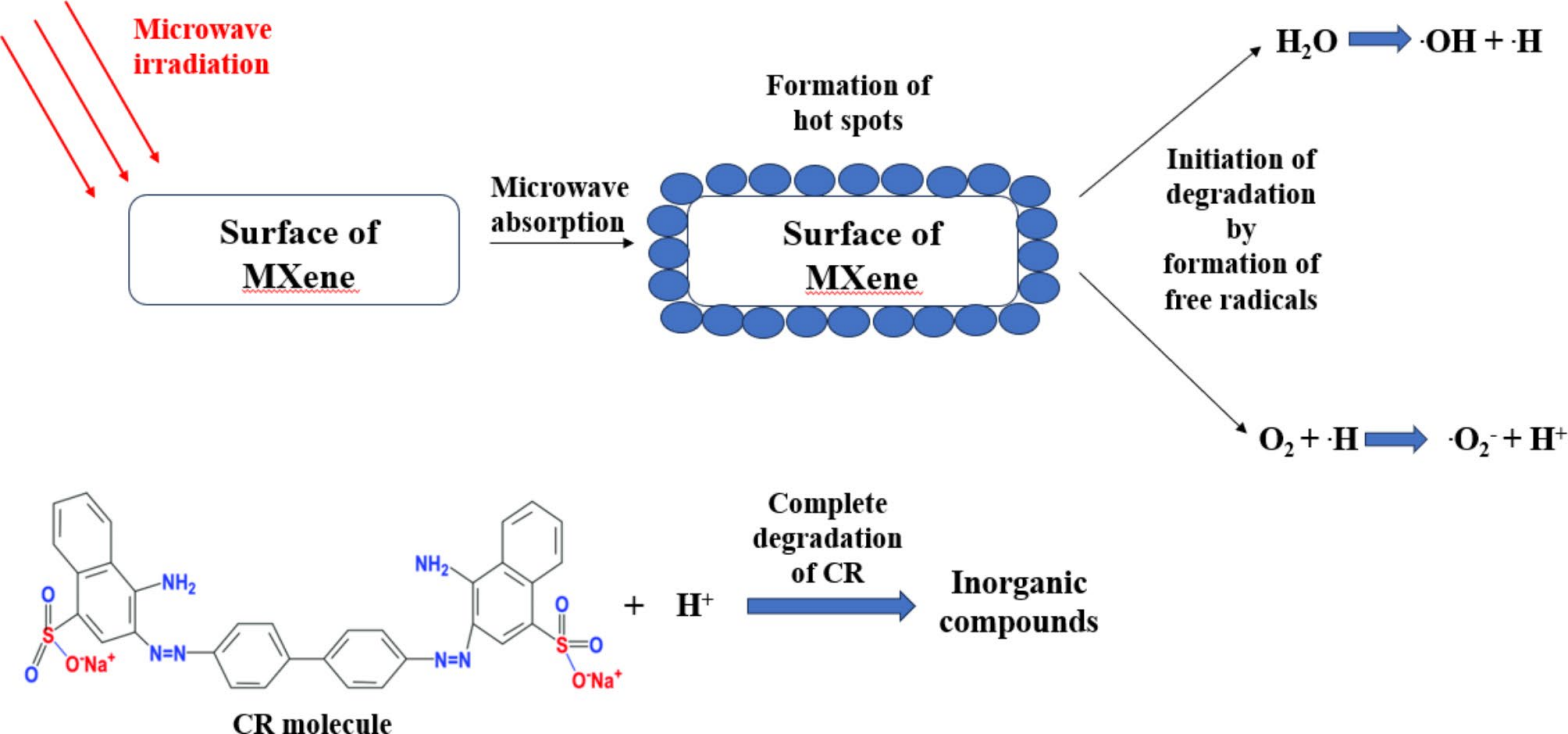
Proposed CR dye degradation mechanism under Microwave irradiation in the presence of 2D Ti_3_C_2_T_x_ Mxene.

Based on the results, 2D Ti_3_C_2_T_x_ MXene is an excellent microwave catalyst with high degradation efficiency for CR dye. From the results mentioned above, the mechanism of the microwave-induced degradation of CR dye could be concluded (Fig. 6). As shown in Fig. 6, when a microwave-absorbing catalyst is exposed to microwave radiation, it will effectively absorb the radiation, and hot spots form on the catalyst’s surface. These hot spots, with temperatures over 1000 °C, are considered active sites where the degradation process starts. Under MW irradiation, the water molecules in the dye solution may be broken down at this high temperature into ^•^OH and ^•^H radicals. Additionally, the oxygen in the mixture may react with ^•^H to generate ^•^O_2_^–^ and .OH after absorbing numerous hot spots^13,52,53^. Moreover, in this system, 2D Ti_3_C_2_T_x_ Mxene can displays catalytic activity in a similar photocatalytic reaction by utilizing the high heat from the hot spots^53^.

The degradation is primarily attributed to the generation of hydroxyl (^•^OH) and hydrogen (^•^H) radicals produced by the microwave heating effect on water. Microwaves induce localized heating and create a thermal non-equilibrium, promoting water molecules’ dissociation. These radicals are highly reactive and attack the pollutant molecules, leading to their breakdown into more minor, less harmful compounds.

The role of Na-EDTA, in this context, is secondary and serves as a chelating agent to bind metal ions, which may otherwise catalyse undesired side reactions. However, its influence on the primary radical-driven degradation pathway is minimal. Key reactions under microwave heating:

Microwave-induced water splitting:

3 $${{\text{H}}_2}{\text{O}}+\text{MW} \to {}^{ \cdot }\text{OH}+{}^{ \cdot }\text{H}$$

Radical-driven pollutant degradation:

4 $${\text{Pollutant}}+{}^{ \cdot }{\text{OH}} \to {\text{Degraded}}\;{\text{products}}$$

When molecular oxygen reacts with hydrogen radicals, it forms superoxide anions (^**·**^O_2_^−^) and protons (H^+^). The reaction can be expressed as:

5 $${\text{O}_2}+{}^{ \cdot }\text{H} \to {}^{ \cdot }\text{O}_{2}^{ - }+{\text{H}^+}$$

### Recyclability of the prepared catalyst

**Fig. 7:**
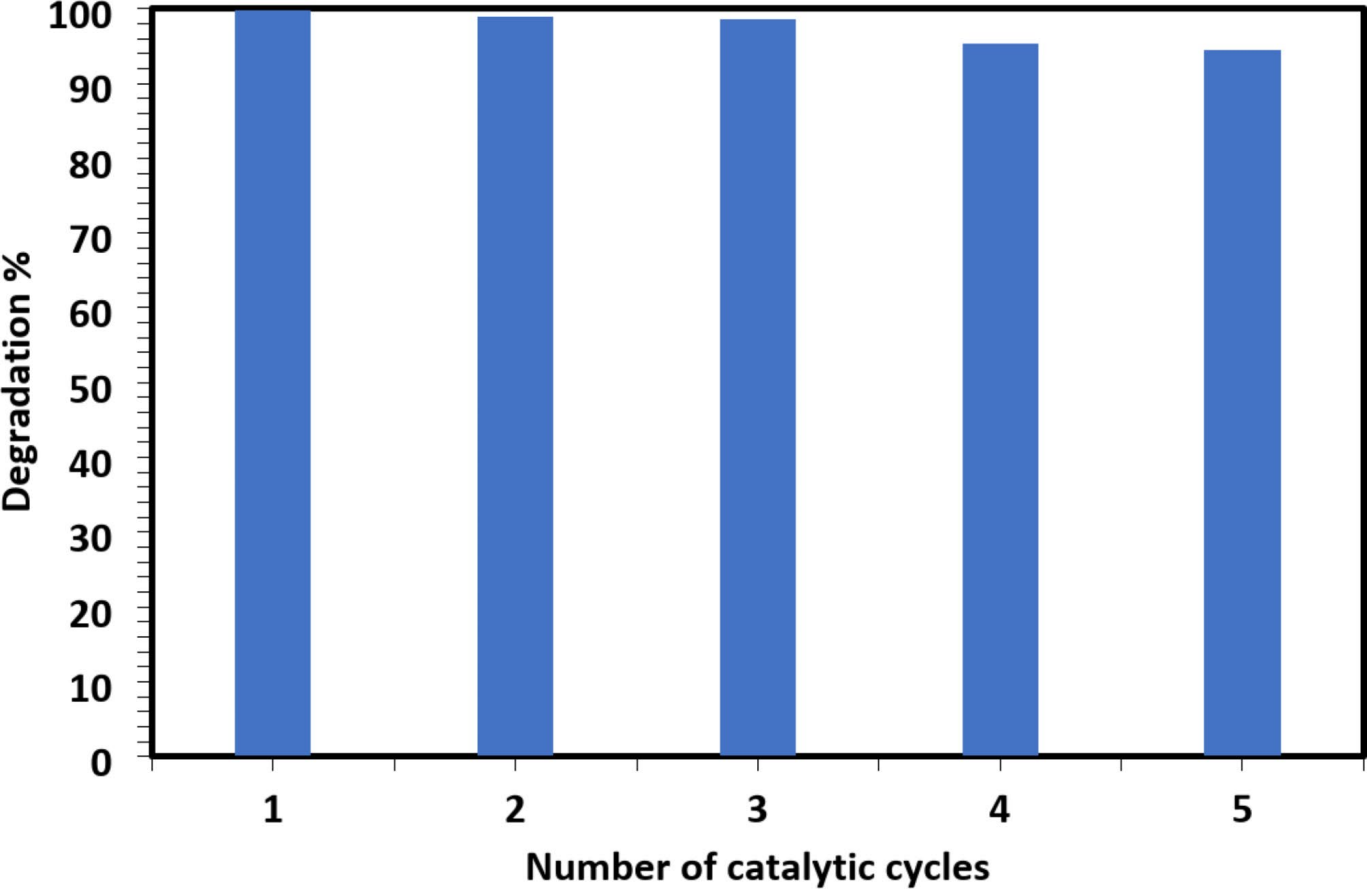
The reusability of the 2D Ti_3_C_2_T_x_ MXene catalyst in CR dye degradation.

Recycling is a crucial factor for any catalyst under test for the profitable and cost-effective removal of organic pollutants. In this work, the synthesized 2D Ti_3_C_2_T_x_ Mxene catalyst was reused five successive times in the degradation of CR dye. The high degrading efficiency was sustained throughout the five catalytic cycles, as illustrated in Fig. 7, suggesting that the catalyst has excellent stability and reusability.

### Comparison with literature

Table 3 displays previous work done on the degradation of CR dye. Different degradation methods, such as photocatalytic degradation, electrochemical degradation, and degradation using microwave irradiation, have been employed to get rid of CR dye in water. Compared to other work, as noticed from Table 3, our work showed the most efficient and less time-consuming method and catalyst.

**Table 3:** Results obtained from previous work concerning CR dye degradation.

Catalyst	Application	Efficiency	Refs.
Magnetic silica-coated Ag_2_WO_4_/Ag_2_S	Photocatalytic degradation of CR under visible light	Photodegradation efficiency of 99.5% against CR dye	^32^
Ni-TiO_2_ nanoflakes	Photocatalytic degradation of CR under UV irradiation	Maximum CR removal percentage was approximately 92.31% in 180 min	^54^
C_3_N_4_/RGO/Bi_2_Fe_4_O_9_	Photocatalytic degradation of CR under visible light	About 87% of CR was degraded in 60 min	^55^
ZnO nanoparticles	Photocatalytic degradation of CR under UV irradiation	99.9% degradation of CR dye within 80 min	^56^
MgAl_2_O_4_ nanoparticles	Photocatalytic degradation of CR under UV irradiation	Maximum degradation was 99.27% within 80 min	^57^
Pt/CuNPs electrode	Electrochemical degradation of the CR dye	Degradation efficiency of 95.95% was achieved in 60 min	33
Biochar supported TiO_2_-g-C_3_N_4_	Photocatalytic degradation of CR under solar irradiation	About 60% degradation efficiency could be achieved after 3 h	^58^
CdFe_2_O_4_ nanoparticles	Microwave-induced catalytic degradation method	Degradation reached 94.4% within 10 min	^59^
2D Ti_3_C_2_T_x_ MXene	Catalytic degradation of CR using microwave irradiation	Complete degradation could almost be achieved in only 6 min	This work

### RSM study

An ANOVA analysis was performed on the model of choice to assess its significance and identify the terms influencing the removal percentage. Table 4 lists the ANOVA analysis results^33–39^. The *F*-values show how vital the factors under study and their interactions are with the chosen response. As the *F*-value surpasses 1, the influence of the associated factor or interaction on the response is more significant. It was discovered that the initial CR dye concentration had the most significant impact on the CR dye elimination percentage based on the numbers displayed in Table 4. The model is essential, as indicated by the model’s *F*-value (155.93) (Table 5). In addition, terms that have *p*-values less than 0.05 are regarded as significant. The model’s *p*-value, which is less than 0.0001, can be seen to indicate its significance. Furthermore, the model’s strength is further demonstrated by the difference between the adjusted *R*^2^ (0.9887) and predicted *R*^2^ (0.9206) is less than 0.2.

Based on the obtained results, the following Eqs. (6, 7) for CR dye removal % were obtained:

6 $$\begin{aligned} \text{Removal} \% \;{\text{for\,coded\,factors}}= & 86.59+14.23\text{A} - 19.22\text{B}+10.81\text{C}+13.70\text{AB} \\ & - 5.75\text{AC}+8.00\text{BC} - 2.47\text{A}{^2} - 5.05\text{B}{^2} - 2.90\text{C}{^2} \\ \end{aligned}$$

7 $$\begin{aligned} \text{Removal}\%\,\text{for\,actual\,factors}= & 45.52+2.30\,\text{Dose} - 1.44\,\text{Conc.}+5.24\,\text{Time}+0.03\,\text{Dose} \times \text{Conc.} \\ & - 0.10\,\text{Dose} \times \text{Time}+0.03\,\text{Conc.} \times \text{Time} \\ &- 0.02\,\text{Dose}{^2} - 0.002\,\text{Conc.}{^2} - 0.08\,\text{Time}{^2} \\ \end{aligned}$$

**Table 4:** Experimental design for CR dye microwave-induced degradation using 2D Ti_3_C_2_T_x_ MXene as a catalyst.

Run	Factor 1 A: Dose (mg)	Factor 2 B: Conc. (mg/L)	Factor 3 C: Time (min)	Experimental removal %	Predicted removal %
1	40	75	10	86.59	86.59
2	40	25	16	99.999	100.67
3	40	75	10	86.59	86.59
4	40	75	10	86.59	86.59
5	30	25	10	99.999	97.77
6	30	125	10	30.734	31.92
7	40	25	4	92.308	95.05
8	50	75	4	91.954	90.39
9	40	125	16	80.963	78.22
10	40	75	10	86.59	86.59
11	50	125	10	85.551	87.78
12	50	25	10	99.999	98.82
13	30	75	4	50.958	50.44
14	40	75	10	86.59	86.59
15	40	125	4	41.284	40.61
16	30	75	16	81.992	83.55
17	50	75	16	99.999	100.51

**Table 5:** *F* and *p*-values determined by Box-Behnken design for influential variables in CR dye degradation.

Source	Sum of Squares	df	Mean Square	F-value	*p*-value	
Model	6834.05	9	759.34	155.93	< 0.0001	**significant**
A-Dose	1619.36	1	1619.36	332.53	< 0.0001	
B-Conc.	2955.75	1	2955.75	606.95	< 0.0001	
C-Time	934.19	1	934.19	191.83	< 0.0001	
AB	751.21	1	751.21	154.26	< 0.0001	
AC	132.13	1	132.13	27.13	0.0012	
BC	255.80	1	255.80	52.53	0.0002	
A²	25.61	1	25.61	5.26	0.0556	
B²	107.52	1	107.52	22.08	0.0022	
C²	35.36	1	35.36	7.26	0.0309	
Residual	34.09	7	4.87			
Lack of Fit	34.09	3	11.36			
Pure Error	0.0000	4	0.0000			
Cor Total	6868.13	16				
Std. Dev.	2.21	R²	0.9950			
Mean	81.69	Adjusted R²	0.9887			
C.V. %	2.70	Predicted R²	0.9206			
Adeq Precision	40.6213					

**Fig. 8:**
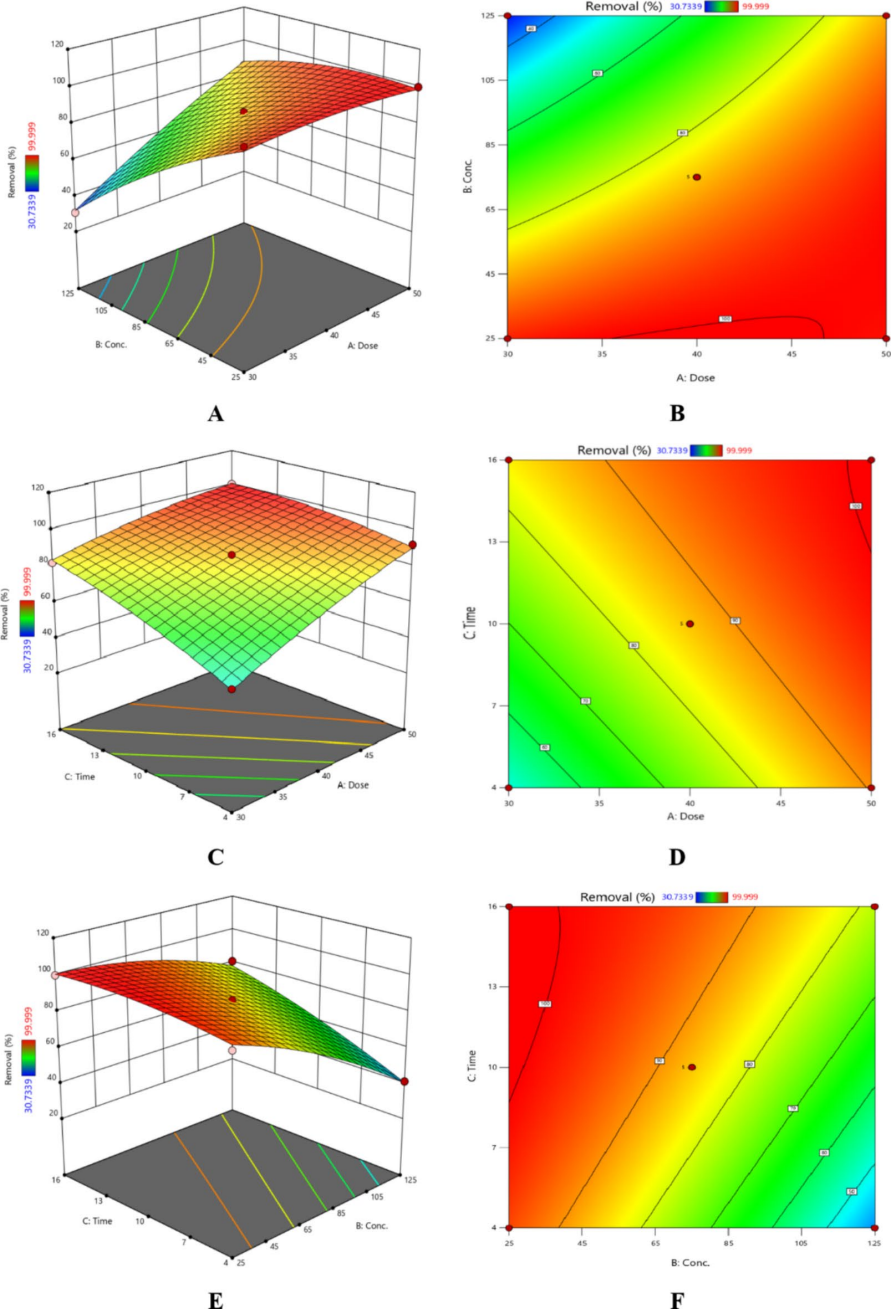
Combined effects of independent variables: (**A**,**B**) Catalyst dose and initial concentration, (**C**,**D**) Catalyst dose and time, and (**E**,**F**) initial concentration and time.

The combined effects of reaction time, catalyst dosage, and initial CR dye concentration on the elimination percentage of CR dye are depicted in Fig. 8. Low CR dye concentrations, high catalyst dosages and delayed reaction periods are ideal for achieving the best removal percentage^40,41^.

**Fig. 9:**
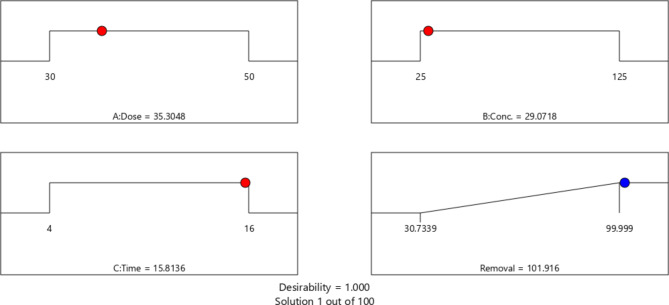
Optimization conditions through CCD settings.

**Fig. 10:**
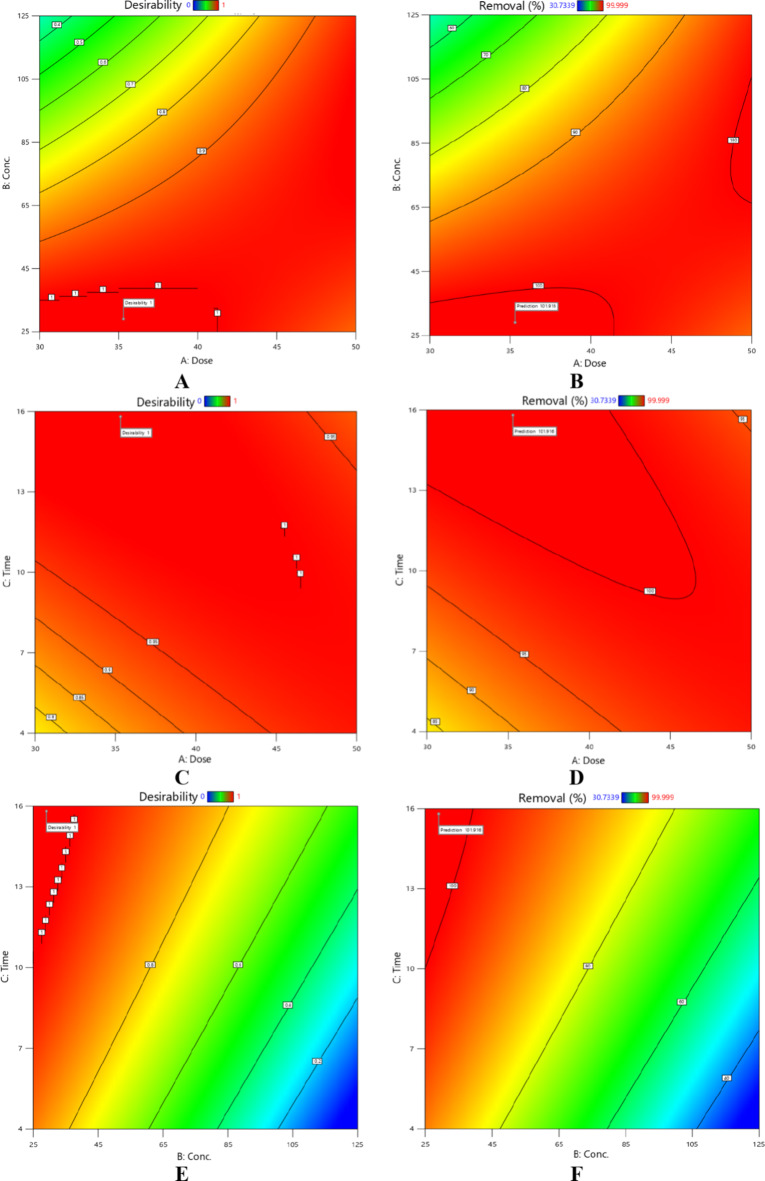
Desired regions for optimal degradation of CR by CCD settings.

As shown in Fig. 9, the ideal operating parameters were numerically determined to yield the most significant CR dye elimination percentage. Figure 10 shows the desired regions for optimal CR dye and desirability contour.

## Conclusion

Herein, the degradation efficiency of 2D Ti_3_C_2_T_x_ MXene in a microwave-induced reaction system was examined using CR dye as the organic pollutant. Results showed that the obtained catalyst exhibited superior catalytic abilities in the microwave-induced degradation of CR dye, and nearly complete degradation (~ 99%) could be attained in only 6 min. In addition, the impact of initial pH (2–10), catalyst dosage (30–50 mg), and initial CR dye concentration (25–125 ppm) on the degradation rate was investigated. All experiments were conducted at the natural pH (6) of CR dye solution = 6.4 because the highest degradation efficiency (99.2%) was obtained at this pH. Large catalyst dosages and low initial CR dye concentrations significantly increased the degradation rate. For example, when employing the highest catalyst dosage (40 mg) and lowest initial CR concentration (25 ppm), a high degradation efficiency of 98.7% was achieved in only 6 min. In addition, the degradation reaction was found to follow the pseudo-first-order kinetic model with values of rate constant ranging from 0.04 to 0.83 min^−1^, depending on the used catalyst dosage and initial CR dye concentration. The synthesized catalyst was highly stable and could be reused more than 5 times without losing its catalytic activity. Moreover, the active species participating in the degradation process were determined using a scavenger test, and the results showed that the dye degradation is primarily attributed to the generation of hydroxyl (**·**OH) and hydrogen (**·**H) radicals, produced by the microwave heating effect on water. Finally, optimization of parameters was conducted using RSM and results concluded that a maximum degradation percentage (101.92%) could be reached when employing 35.30 mg of the catalyst and 29.07 ppm of CR solution in 15.81 min.

## Electronic Supplementary Material

Below is the link to the electronic supplementary material.

Supplementary Material 1

## Data Availability

The datasets used in this investigation are accessible for review upon request from the paper’s corresponding author.

## References

[1] Le, A. T. et al. Immobilization of zinc oxide-based photocatalysts for organic pollutant degradation: A review. *J. Environ. Chem. Eng.* **10**(5), 108505 (2022).

[2] Chen, D. et al. Photocatalytic degradation of organic pollutants using TiO2-based photocatalysts: A review. *J. Clean. Prod.* **268**, 121725 (2020).

[3] Wang, X. et al. Structure and electromagnetic properties of Ti3C2Tx MXene derived from Ti3AlC2 with different microstructures. *Ceram. Int.* **47**(10), 13628–13634 (2021).

[4] Zhu, D. & Zhou, Q. *Action and mechanism of semiconductor photocatalysis on degradation of organic pollutants in water treatment: A review* 100255 (Environmental Nanotechnology, Monitoring & Management, 2019).

[5] Zhang, X. et al. Functionalized metal-organic frameworks for photocatalytic degradation of organic pollutants in environment. *Chemosphere* **242**, 125144 (2020). 31669994 10.1016/j.chemosphere.2019.125144

[6] Anandan, S., Ponnusamy, V. K. & Ashokkumar, M. A review on hybrid techniques for the degradation of organic pollutants in aqueous environment. *Ultrason. Sonochem.* **67**, 105130 (2020). 32315972 10.1016/j.ultsonch.2020.105130

[7] Yang, Y. et al. Chitosan-capped ternary metal selenide nanocatalysts for efficient degradation of Congo red dye in sunlight irradiation. *Int. J. Biol. Macromol.* **167**, 169–181 (2021). 33249161 10.1016/j.ijbiomac.2020.11.167

[8] Iark, D. et al. Enzymatic degradation and detoxification of azo dye Congo red by a new laccase from Oudemansiella canarii. *Bioresour. Technol.* **289**, 121655 (2019). 31247524 10.1016/j.biortech.2019.121655

[9] Liu, J. et al. Adsorption of Congo red dye on FexCo3-xO4 nanoparticles. *J. Environ. Manage.* **238**, 473–483 (2019). 30877940 10.1016/j.jenvman.2019.03.009

[10] Zheng, Y. et al. Hierarchical flower-like nickel (II) oxide microspheres with high adsorption capacity of Congo red in water. *J. Colloid Interface Sci.* **504**, 688–696 (2017). 28622562 10.1016/j.jcis.2017.06.014

[11] Aoopngan, C. et al. Amine-functionalized and hydroxyl-functionalized magnesium ferrite nanoparticles for Congo red adsorption. *ACS Appl. Nano Mater.* **2**(8), 5329–5341 (2019).

[12] Han, L. J. et al. Effective adsorption of Congo red by a MOF-based magnetic material. *Dalton Trans.* **48**(14), 4650–4656 (2019). 30892337 10.1039/c9dt00813f

[13] Gao, J. et al. Rapid degradation of azo dye Direct Black BN by magnetic MgFe2O4-SiC under microwave radiation. *Appl. Surf. Sci.* **379**, 140–149 (2016).

[14] Zhang, L. et al. Investigation on the degradation of brilliant green induced oxidation by NiFe2O4 under microwave irradiation. *Chem. Eng. J.* **173**(3), 737–742 (2011).

[15] Fang, X. et al. Investigation on microwave absorbing properties of loaded MnFe2O4 and degradation of reactive brilliant red X-3B. *Appl. Catal. B* **162**, 544–550 (2015).

[16] Zhang, L. et al. Investigation on the degradation of acid fuchsin induced oxidation by MgFe2O4 under microwave irradiation. *J. Mol. Catal. A: Chem.* **335**(1–2), 31–37 (2011).

[17] Zhang, Z. et al. Investigation on the rapid degradation of congo red catalyzed by activated carbon powder under microwave irradiation. *J. Hazard. Mater.* **147**(1–2), 325–333 (2007). 17293037 10.1016/j.jhazmat.2006.12.083

[18] Olmedo, L., Hourquebie, P. & Jousse, F. Microwave absorbing materials based on conducting polymers. *Adv. Mater.* **5**(5), 373–377 (1993).

[19] Zhou, X. et al. Broadband high-performance microwave absorption of the single-layer Ti3C2Tx MXene. *J. Mater. Sci. Technol.* **115**, 148–155 (2022).

[20] Yan, H. et al. Facile constructing Ti3C2Tx/TiO2@ C heterostructures for excellent microwave absorption properties. *J. Colloid Interface Sci.* **654**, 1483–1491 (2024). 37867074 10.1016/j.jcis.2023.10.076

[21] Mudasar, M. et al. Tailoring permittivity and permeability of M-type hexagonal ferrite and 2D Ti3C2Tx MXene composites for broadband microwave stealth performance. *Mater. Chem. Phys.* 129031 (2024).

[22] Zhang, X. et al. Novel solvothermal preparation and enhanced microwave absorption properties of Ti3C2Tx MXene modified by in situ coated Fe3O4 nanoparticles. *Appl. Surf. Sci.* **484**, 383–391 (2019).

[23] Yan, H. et al. Dielectric–magnetic synergistic design of Ti3C2Tx@ C/NiZn ferrite composite for effective microwave absorption performance. *Appl. Surf. Sci.* **633**, 157602 (2023).

[24] Zhang, Z. et al. The recent progress of MXene-Based microwave absorption materials. *Carbon* **174**, 484–499 (2021).

[25] Zeng, X. et al. Construction of NiCo2O4 nanosheets-covered Ti3C2Tx MXene heterostructure for remarkable electromagnetic microwave absorption. *Carbon* **193**, 26–34 (2022).

[26] Fan, X. et al. Elaborately designed 3D honeycomb M – Ti3C2Tx@ MoS2@ C heterostructures as advanced microwave absorbers. *Appl. Surf. Sci.* **625**, 157116 (2023).

[27] Cai, Z. et al. Ti3C2Tx MXene/graphene oxide/Co3O4 nanorods aerogels with tunable and broadband electromagnetic wave absorption. *Chem. Eng. J.* **462**, 142042 (2023).

[28] Lei, Y. et al. A broadband and strong microwave absorption of Ti3C2Tx MXene/PPy composites with a core-shell structure. *Synth. Met.* **293**, 117254 (2023).

[29] Chang, M. et al. Tuning microwave absorption properties of Ti3C2Tx MXene-based materials: Component optimization and structure modulation. *J. Mater. Sci. Technol.* **148**, 150–170 (2023).

[30] Rao, H. et al. Electrostatically self-assembled hierarchical magnetic Co7Fe3@ C/Ti3C2Tx nanocomposite for high-efficient microwave absorption. *Mater. Today Phys.* **41**, 101355 (2024).

[31] Yadav, P. et al. Enhanced degradation of Congo-red dye by Cr3 + doped α-Fe2O3 nano-particles under sunlight and industrial wastewater treatment. *Chemosphere* **343**, 140208 (2023). 37739127 10.1016/j.chemosphere.2023.140208

[32] Jabbar, Z. H. et al. Photocatalytic degradation of Congo red dye using magnetic silica-coated Ag2WO4/Ag2S as Type I heterojunction photocatalyst: Stability and mechanisms studies. *Mater. Sci. Semiconduct. Process.* **153**, 107151 (2023).

[33] Ganash, A. et al. Efficient electrochemical degradation of congo red dye by Pt/CuNPs electrode with its attractive performance, energy consumption, and mechanism: Experimental and theoretical approaches. *J. Water Process. Eng.* **56**, 104497 (2023).

[34] Riyanti, F. et al. Photocatalytic degradation of methylene blue and Congo red dyes from aqueous solutions by bentonite-Fe3O4 magnetic. *Commun. Sci. Technol.* **8**(1), 1–9 (2023).

[35] Meky, A. I., Hassaan, M. A., Fetouh, H. A., Ismail, A. M. & Nemr, E. A., Hydrothermal fabrication, characterization and RSM optimization of cobalt-doped zinc oxide nanoparticles for antibiotic photodegradation under visible light. *Sci. Rep.* **14**(1), 2016 (2024). 10.1038/s41598-024-52430-8 PMC11231344 38263230

[36] Hassaan, M. A., Meky, A. I., Fetouh, H. A., Ismail, A. M. & Nemr, E. A., Central composite design and mechanism of antibiotic ciprofloxacin photodegradation under visible light by green hydrothermal synthesized cobalt-doped zinc oxide nanoparticles. *Sci. Rep.* **14**(1), 9144 (2024). 10.1038/s41598-024-58961-4 PMC11551219 38644378

[37] Hassaan, M. A. et al. Synthesis, characterization, optimization and application of Pisum sativum peels S and N-doping biochars in the production of biogas from Ulva lactuca. *Renew. Energy* **221**, 119747 (2024).

[38] Hassaan, M. A. et al. A., Box-Behnken design and life cycle assessment for nickel oxide nanoparticles application in biomethane production. *Chem. Eng. J.* **474**, 145924 (2023).

[39] Hassaan, M. A. et al. Application of multi-heteroatom doping biochar in a newly proposed mechanism of electron transfer in biogas production. *Chem. Eng. J.* **470**, 144229 (2023).

[40] Meky, A. I., Hassaan, M. A., Fetouh, H. A., Ismail, A. M. & Nemr, E. A., Cube-shaped Cobalt-doped zinc oxide nanoparticles with increased visible-light-driven photocatalytic activity achieved by green co-precipitation synthesis. *Sci. Rep.* **13**(1), 19329 (2023). 10.1038/s41598-023-46464-7 PMC10630306 37935868

[41] Ragab, S., Elkatory, M. R., Hassaan, M. A. & El Nemr, A. Experimental, predictive and RSM studies of H2 production using Ag-La-CaTiO3 for water-splitting under visible light. *Sci. Rep.* **14**(1), 1019 (2024). 10.1038/s41598-024-51219-z PMC10781765 38200036

[42] Hassaan, M. A. et al. Improved methylene blue adsorption from an aqueous medium by ozone-triethylenetetramine modification of sawdust-based biochar. *Sci. Rep.* **13**(1), 12431 (2023). 10.1038/s41598-023-39495-7 PMC10394039 37528164

[43] Hassaan, M. A. & El Nemr, A. Classification and identification of different minerals in the Mediterranean sediments using PSA, FTIR, and XRD techniques. *Mar. Polluti. Bull.* **173**, 113070 (2021). 10.1016/j.marpolbul.2021.113070 34678547

[44] Eldeeb, T. M. et al. Adsorption of methylene blue (MB) dye on ozone, purified and sonicated sawdust biochars. *Biomass Convers. Biorefinery*. **14**(8), 9361–9383 (2024).

[45] Hassaan, M. A. et al. A., Isotherm and kinetic investigations of sawdust-based biochar modified by ammonia to remove methylene blue from water. *Sci. Rep.* **13**(1), 12724 (2023). 10.1038/s41598-023-39971-0 PMC10404293 37543711

[46] Eleryan, A. et al. Copper (II) ion removal by chemically and physically modified sawdust biochar. *Biomass Convers. Biorefin.* **14**(8), 9283–9320 (2024).

[47] Eleryan, A. et al. Mandarin Biochar-TETA (MBT) prepared from Citrus reticulata peels for adsorption of Acid Yellow 11 dye from water. *Sci. Rep.* **12**(1), 17797 (2022). 10.1038/s41598-022-22359-x PMC9587999 36273033

[48] Eldeeb, T. M. et al. Biosorption of Acid Brown 14 dye to mandarin-CO-TETA derived from mandarin peels. *Biomass Convers. Biorefin* 1–21 (2022).

[49] Eleryan, A. et al. Kinetic and isotherm studies of acid orange 7 dye absorption using sulphonated mandarin biochar treated with TETA. *Biomass Convers. Biorefinery*. **14**(9), 10599–10610 (2024).

[50] El-Nemr, M. A. et al. Adsorption of Cr6 + ion using activated Pisum sativum peels-triethylenetetramine. *Environ. Sci. Pollut. Res.* **29**(60), 91036–91060 (2022). 10.1007/s11356-022-21957-6 PMC9722890 35881295

[51] El-Nemr, M. A. et al. The use of biochar-NH2 produced from watermelon peels as a natural adsorbent for the removal of Cu(II) ions from water. Biomass Convers. Biorefin. (2022).

[52] Shen, M. et al. Microwave hydrothermal-assisted preparation of novel spinel-NiFe2O4/natural mineral composites as microwave catalysts for degradation of aquatic organic pollutants. *J. Hazard. Mater.* **350**, 1–9 (2018). 29448208 10.1016/j.jhazmat.2018.02.014

[53] Liu, S. et al. Anchoring Fe3O4 nanoparticles on carbon nanotubes for microwave-induced catalytic degradation of antibiotics. *ACS Appl. Mater. Interfaces*. **10**(35), 29467–29475 (2018). 30091894 10.1021/acsami.8b08280

[54] Indira, K. et al. Photocatalytic degradation of congo red dye using nickel–titanium dioxide nanoflakes synthesized by Mukia madrasapatna leaf extract. *Environ. Res.* **202**, 111647 (2021). 34237334 10.1016/j.envres.2021.111647

[55] Shekardasht, M. B. et al. Preparation of a novel Z-scheme g-C3N4/RGO/Bi2Fe4O9 nanophotocatalyst for degradation of Congo Red dye under visible light. *Diam. Relat. Mater.* **109**, 108008 (2020).

[56] Gaur, J. et al. Photocatalytic degradation of Congo red dye using zinc oxide nanoparticles prepared using Carica papaya leaf extract. *Mater. Today Sustain.* **22**, 100339 (2023).

[57] Al-Farraj, E. S. & Abdelrahman, E. A. Efficient photocatalytic degradation of congo red dye using facilely synthesized and characterized MgAl2O4 nanoparticles *ACS Omega* (2024). 10.1021/acsomega.3c08485 PMC10831849 38313534

[58] Patil, A. et al. Walnut shell biochar supported TiO2–g-C3N4 heterojunction photocatalyst for solar photocatalytic degradation of congo red. *Biomass Convers. Biorefin.* **13**(11), 9537–9547 (2023).

[59] Shi, W. et al. Magnetic nano-sized cadmium ferrite as an efficient catalyst for the degradation of Congo red in the presence of microwave irradiation. *RSC Adv.* **5**(63), 51027–51034 (2015).

