# Supplementary material for: Microwave-induced degradation of Congo red dye in the presence of 2D Ti3C2Tx MXene as a catalyst
Source: Sci Rep. 2025 Jan 3;15:634. doi: 10.1038/s41598-024-82911-9 (PMC11698827; doi:10.1038/s41598-024-82911-9)
Supplement: Supplementary file 1 — Supplementary Material 1 [file 41598_2024_82911_MOESM1_ESM.docx]

**Supporting Materials**

**Microwave-induced degradation of Congo red dye in the presence of 2D Ti_3_C_2_T_x_ MXene as a catalyst**

Salma M. El-Mas^1^, Mohamed A. Hassaan^2^, Gehan M. El-Subruiti^1^, Abdelazeem S. Eltaweil^1^, Ahmed El Nemr^2^*

^1^Department of Chemistry, Faculty of Science, Alexandria University, Alexandria, Egypt

^2^Environment Division, National Institute of Oceanography and Fisheries (NIOF), Kayet Bey, Elanfoushy, Alexandria, Egypt

Email: [mhss95@mail.com](mailto:mhss95@mail.com) (M.A. Hassaan); [salma.elmas@alexu.edu.eg](mailto:salma.elmas@alexu.edu.eg) (S.M. El-Mas); [gehanmsubruiti@alexu.edu.eg](mailto:gehanmsubruiti@alexu.edu.eg) (G.M. El-Subruiti); [abdelazeemeltaweil@alexu.edu.eg](mailto:abdelazeemeltaweil@alexu.edu.eg) (A.S. Eltaweil)

*Corresponding author: [ahmedmoustafaelnemr@yahoo.com](mailto:ahmedmoustafaelnemr@yahoo.com); [ahmed.m.elnemr@gmail.com](mailto:ahmed.m.elnemr@gmail.com)

**Figure S1**. CR dye chemical structure (Chemical Formula: C_32_H_22_N_6_Na_2_O_6_S_2_, Molecular Weight: 696.66, and Elemental Analysis: C, 55.17; H, 3.18; N, 12.06; Na, 6.60; O, 13.78; S, 9.20).

***EDX analysis***

The EDX spectra of the precursor Ti_3_AlC_2_ MAX phase and the generated Ti_3_C_2_T_x_ MXene are displayed in Figure S2. Three elements are visible to be present in the precursor MAX phase: Ti, Al, and C. Meanwhile, Ti, C, F and O make up the majority of the prepared 2D Ti_3_C_2_T_x_ MXene, confirming the removal of almost all Al present [1]. The mass percentages of the various components found in Ti_3_AlC_2_ MAX phase and 2D Ti_3_C_2_T_x_ MXene are shown in Figure S2.

| 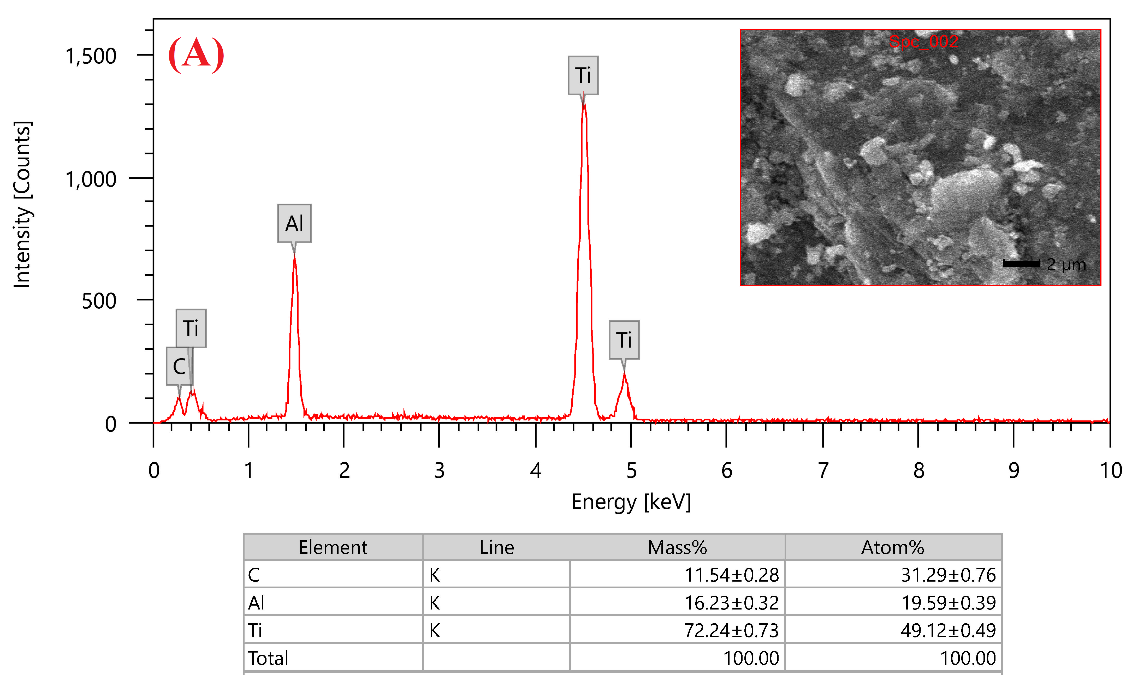 |
| --- |
| 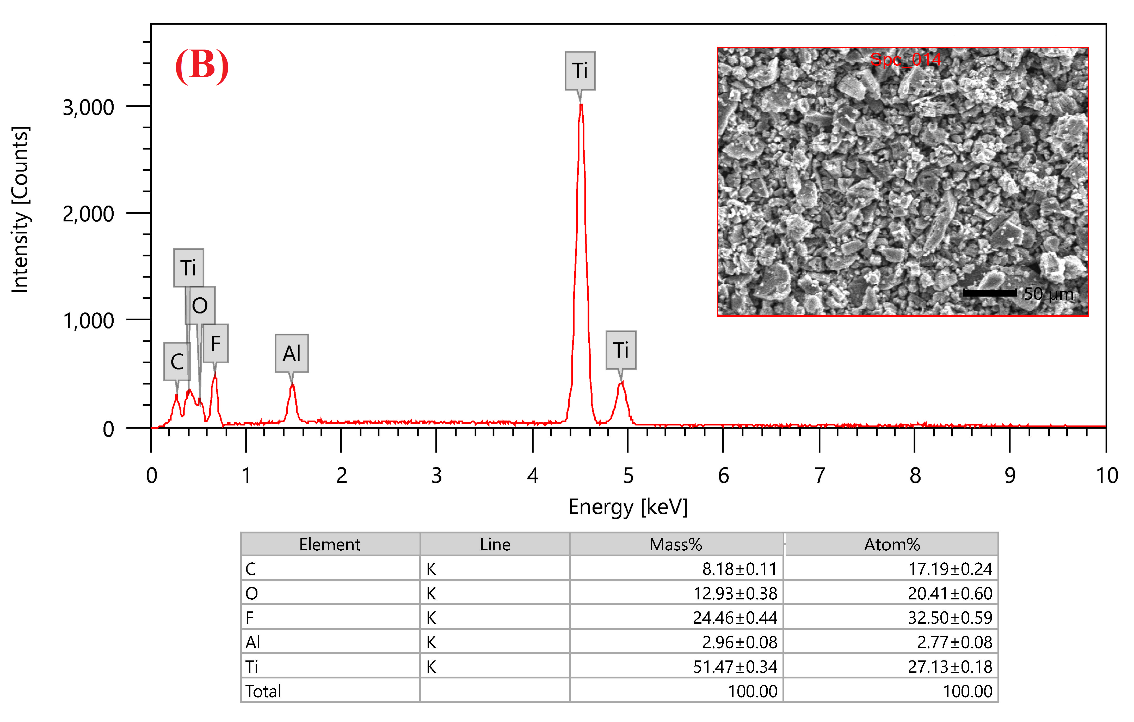 |

**Figure S2**. The EDX spectrum and elemental composition of (a) the precursor Ti_3_AlC_2_ MAX phase and (b) the prepared 2D Ti_3_C_2_T_x_ MXene.

***XRD analysis***

To investigate the phase structure and crystallinity of the precursor Ti_3_AlC_2_ MAX phase and the synthesized 2D Ti_3_C_2_T_x_ MXene, XRD analysis was carried out. Sharp diffraction peaks in the XRD pattern of Ti_3_AlC_2_ MAX phase suggest high phase purity and crystallinity, and they also demonstrate good agreement with the standard pattern of JCPDS 52-0875. Following the etching procedure and the creation of 2D Ti_3_C_2_T_x_ MXene, the distinctive sharp peak of the MAX phase at 39° nearly disappeared, indicating that the Al layers in Ti_3_AlC_2_ MAX have been successfully removed and that 2D layered Ti_3_C_2_T_x_ MXene has been formed (Figure S3) [1-4].

| 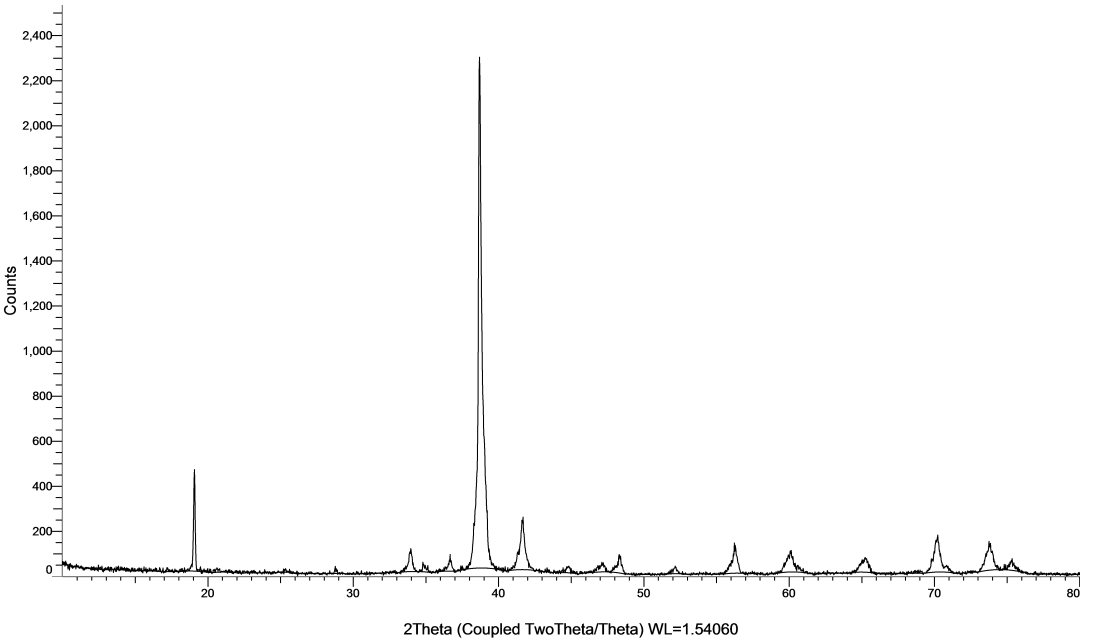  **a** | 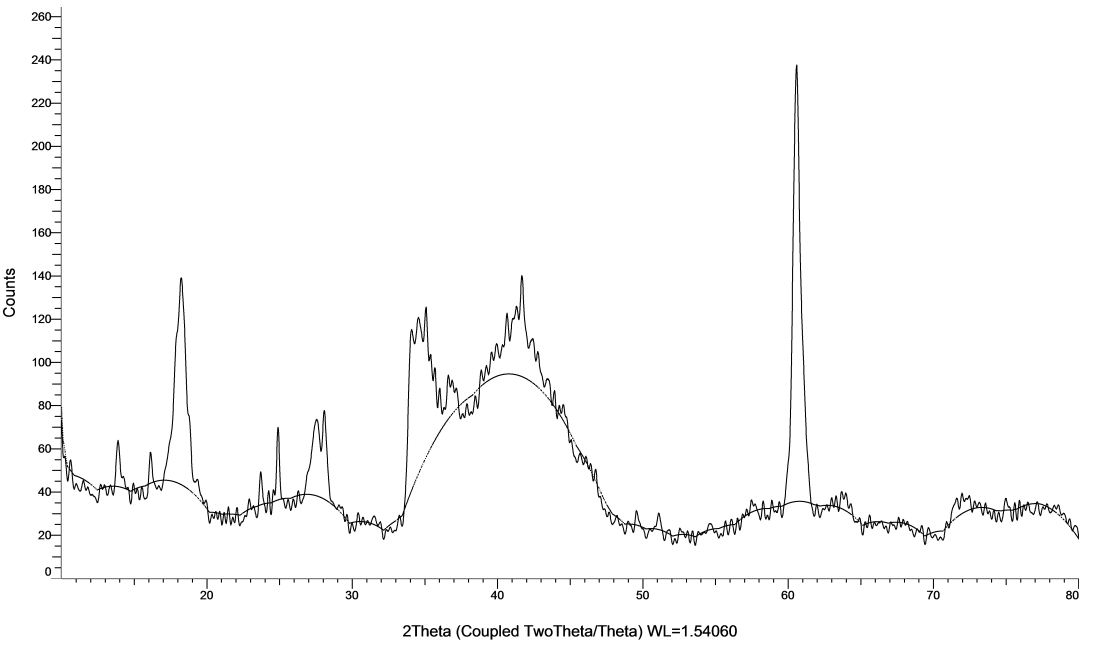  **B** |
| --- | --- |

**Figure S3**. XRD patterns of (a) Ti_3_AlC_2_ MAX and (b) 2D Ti_3_C_2_T_x_ MXene.

***BET surface area analysis***

The surface area of the Ti_3_AlC_2_ MAX phase and synthesized 2D Ti_3_C_2_T_x_ MXene was determined using BET method. Results obtained from BET analysis are recorded in Table S1. It could be seen that the surface area of the synthesized 2D Ti_3_C_2_T_x_ MXene (17.914 m^2^/g) is larger than that of the precursor Ti_3_AlC_2_ MAX (3.615 m^2^/g). This could be attributed to the layered accordion-like structure of 2D Ti_3_C_2_T_x_ MXene [5,6].

**Table S1**. Surface properties of Ti_3_AlC_2_ MAX phase and Ti_3_C_2_T_x_ MXene obtained from BET measurements.

| **Compound** | **S_BET_ (m^2^/g)** | **Pore size (nm)** | **Total pore volume (cm^3^/g)** |
| --- | --- | --- | --- |
| Ti_3_AlC_2_ MAX | 3.615 | 16.073 | 0.0145 |
| 2D Ti_3_C_2_T_x_ MXene | 17.914 | 11.052 | 0.0495 |

***X-ray photoelectron spectroscopy analysis***

The surface elemental compositions and binding energies of the Ti_3_AlC_2_ MAX phase and synthesized 2D Ti_3_C_2_T_x_ MXene were determined by performing XPS analysis. Figure S4 illustrates the XPS spectra of the MAX phase and 2D Ti_3_C_2_T_x_ MXene. As for the precursor MAX phase, survey scans, shown in Figure S4A, indicated the existence of O, Ti, C and Al. The signals of O 1s peak in the survey spectra of the precursor could be assigned to exposure to ambient air [7]. As observed in Figure S4B, the Al peaks decreased significantly indicating the formation of the corresponding 2D Ti_3_C_2_T_x_ MXene upon etching process using HF acid [8]. In addition, the signals of O and F peaks with atomic concentrations of 28.49 and 21.73 %, respectively, indicates the existence of generous amounts of hydroxyl (–OH), oxide (–O) and fluoride (–F) termination groups on the surface of the synthesized 2D Ti_3_C_2_T_x_ MXene [9]. It could be seen that the results of XPS are consistent with those obtained from the elemental EDX analysis.

| 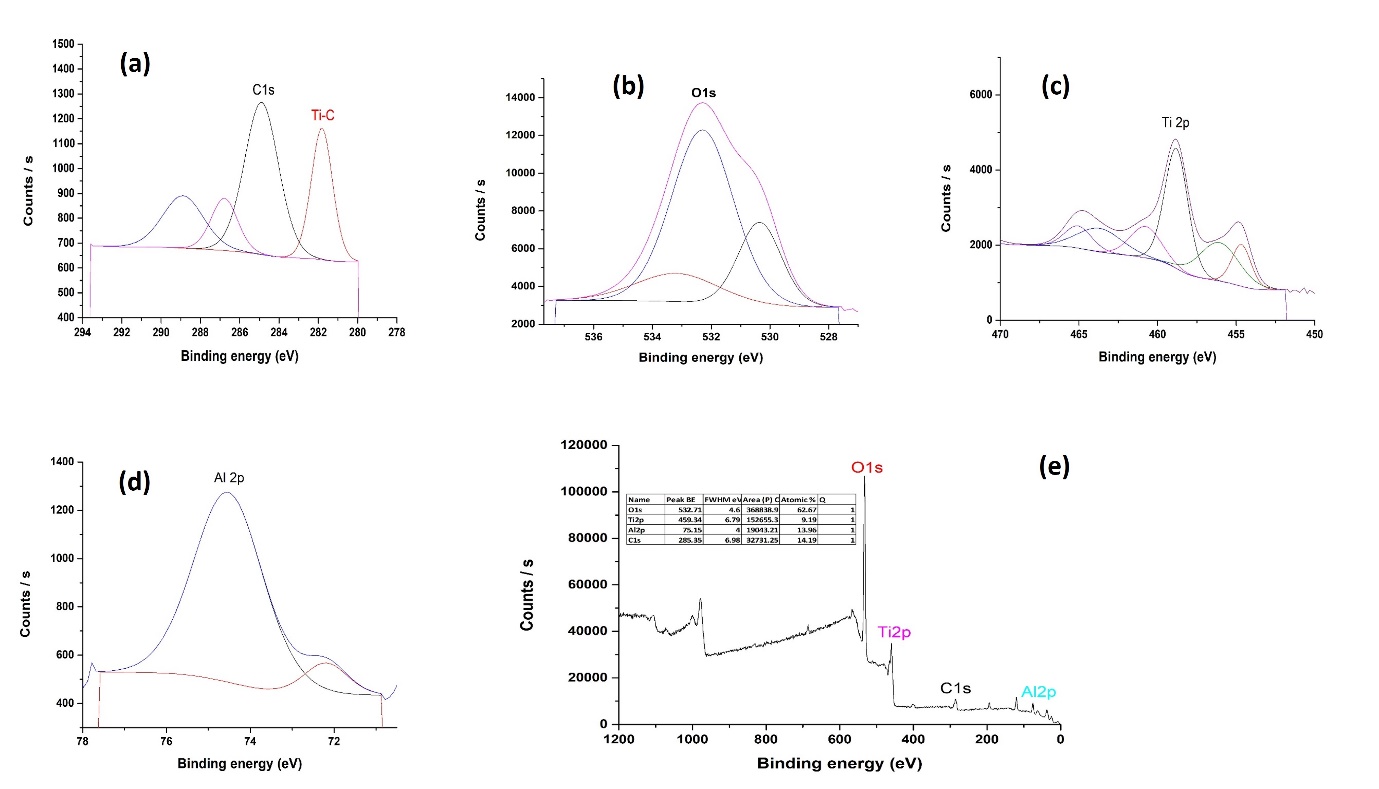 |
| --- |
| **A** |
| 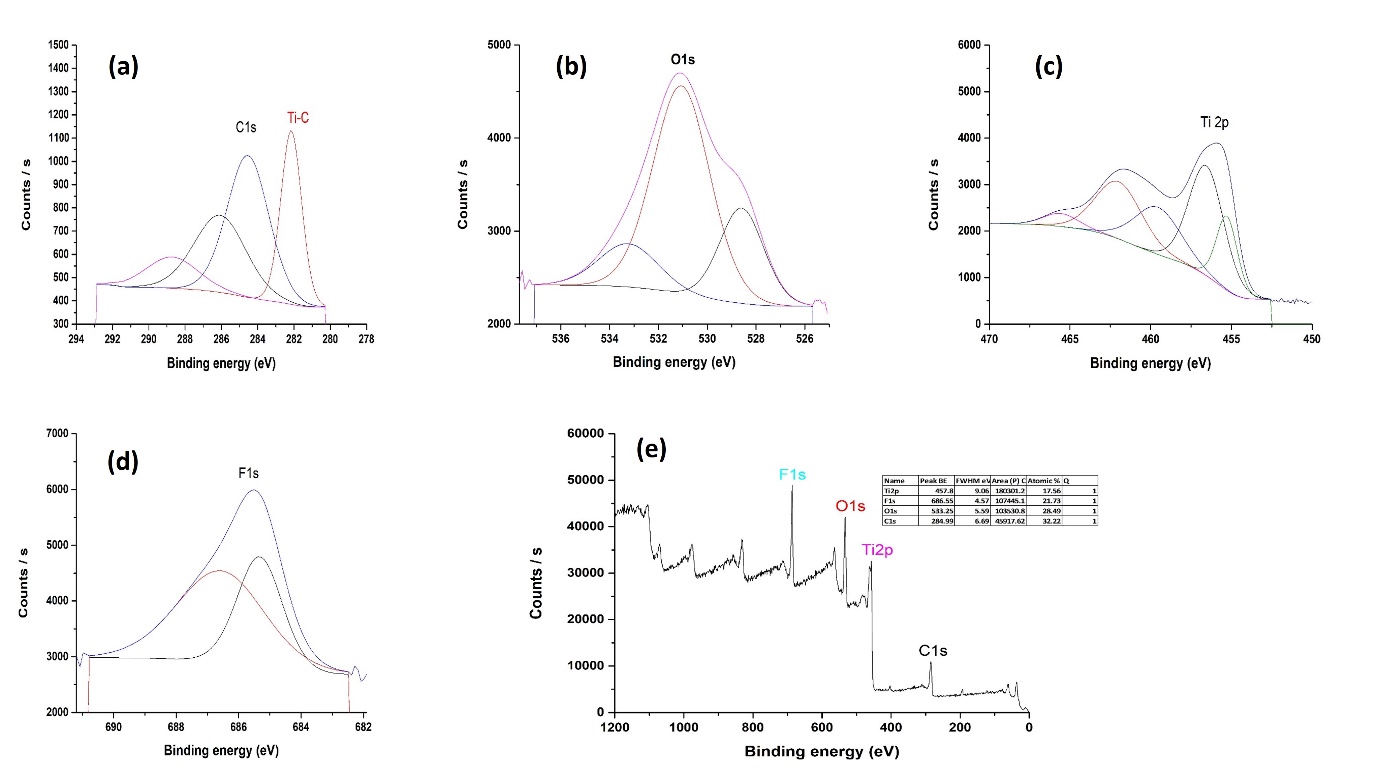 |
| **B** |

**Figure S4**. XPS spectra of (A) Ti_3_AlC_2_ MAX phase, and (B) 2D Ti_3_C_2_T_x_ MXene.

High-resolution scans were also performed for the elements mentioned above in the precursor MAX phase and synthesized 2D Ti_3_C_2_T_x_ MXene. The high resolution XPS scans for C 1s in Ti_3_AlC_2_ MAX phase were measured in the binding energy range of 280-292 eV, as shown in Table S2 and Figure S4. Four sharp peaks were observed at binding energies of approximately 281.8, 284.9, 286.8 and 288.9 eV, corresponding to Ti–C, CH_x_, C–O and C=O respectively [10]. Figure S4 demonstrates fitted peaks of Ti 2p core level over a binding energy range of approximately 450-470 eV, which confirms the presence of Ti as Ti–C in Ti_3_AlC_2_. The high-resolution XPS spectrum of Al 2p, as observed in Figure S4, confirms the presence of metallic Al and Al_2_O_3_ peaks. The reason behind the appearance of these peaks could be due to performing experiments in an open atmosphere. As for Ti_3_C_2_T_x_, from the high-resolution scans of C 1s shown in Figure S4, it could be noted that there is no much difference from those of Ti_3_AlC_2_. Figure S4 depicts the high-resolution spectrum of Ti 2p, showing a positive shift towards higher binding energies indicating an improved bond strength for surface Ti atoms due to the presence of surface functional groups on MXene [11].

**Table S2.** Parameters obtained from the curve fitting of C 1s, Al 2p, Ti 2p, O 1s and F 1s for the MAX phase and 2D Ti_3_C_2_T_x_ MXene.

| **Phase** | **Region** | **BE (eV)** | **FWHM (eV)** | **Atomic %** | **Assignment** |
| --- | --- | --- | --- | --- | --- |
| **Ti_3_AlC_2_ MAX** | C 1s | 281.8 | 1.27 | 24.3 | Ti–C |
|  |  | 284.9 | 2.04 | 45.1 | C–C |
|  |  | 286.8 | 1.57 | 11.9 | C–O |
|  |  | 288.9 | 2.45 | 18.7 | C=O |
|  | Al 2p | 72.19 | 1.21 | 9.19 | Ti-Al |
|  |  | 74.53 | 1.94 | 90.81 | Al_2_O_3_ |
|  | Ti 2p | 454.66 | 1.44 | 10.14 |  |
|  |  | 456.07 | 2.95 | 19.2 |  |
|  |  | 458.81 | 1.74 | 35.27 |  |
|  |  | 460.75 | 2.29 | 12.2 |  |
|  |  | 464.14 | 3.29 | 15.89 |  |
|  |  | 464.86 | 1.46 | 3.84 |  |
|  |  | 471.49 | 3 | 3.46 |  |
|  | O 1s | 530.35 | 1.73 | 21.56 | TiO_2_ |
|  |  | 532.29 | 2.50 | 64.27 | Al_2_O_3_ |
|  |  | 533.14 | 3.37 | 14.17 | Adsorbed H_2_O |
| **2D Ti_3_C_2_T_x_**  **MXene** | C 1s | 282.15 | 1.35 | 23.81 | Ti–C |
|  |  | 284.54 | 2.71 | 39.09 | C–C |
|  |  | 286.07 | 3.37 | 26.4 | C–O |
|  |  | 288.71 | 3.37 | 10.71 | C=O |
|  | Ti 2p | 455.28 | 1.53 | 14.57 |  |
|  |  | 456.55 | 2.55 | 35.95 |  |
|  |  | 459.55 | 3.37 | 20.38 |  |
|  |  | 461.99 | 3.37 | 24.81 |  |
|  |  | 465.55 | 2.55 | 4.29 |  |
|  | O 1s | 530.40 | 2.14 | 22.68 | TiO_2_ |
|  |  | 532.86 | 2.75 | 63.57 | Al_2_O_3_ |
|  |  | 535.04 | 2.86 | 13.75 | Adsorbed H_2_O |
|  | F 1s | 685.32 | 1.72 | 38.61 | F–Ti |
|  |  | 686.55 | 3.38 | 61.39 | AlF_x_ or SiF_x_ |

***SEM and TEM analysis***

The morphology and particle size distribution of the precursor Ti_3_AlC_2_ MAX phase and 2D Ti_3_C_2_T_x_ MXene catalyst were analysed by SEM and TEM instruments. Figure S5 shows the SEM images of the precursor Ti_3_AlC_2_ MAX phase and Ti_3_C_2_T_x_ MXene catalyst. The SEM pictures showed that the precursor Ti_3_AlC_2_ had a dense layered structure, which was etched into a two-dimensional layered accordion-like structure. For Ti_3_AlC_2_ and Ti_3_C_2_T_x_, the respective particle size ranges were roughly 15.41-19.05 and 18.68-23.92 nm. Figure S6 shows the TEM images of the MAX phase and prepared 2D Ti_3_C_2_T_x_ MXene. The images confirm the compact structure of the MAX phase and multi-layered structure of 2D Ti_3_C_2_T_x_ MXene with obvious gaps between layers [12-14].

| 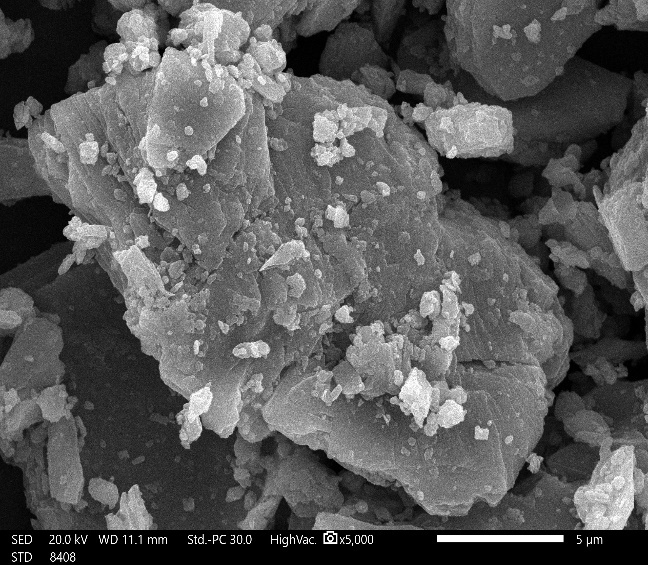  **A** | 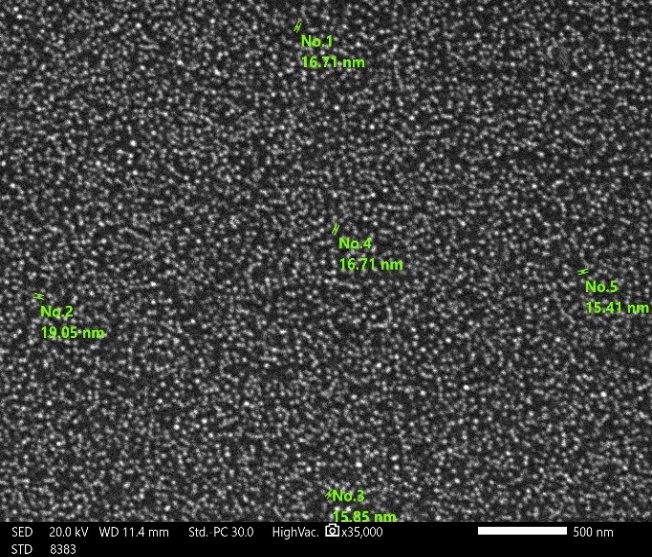  **B** |
| --- | --- |
| 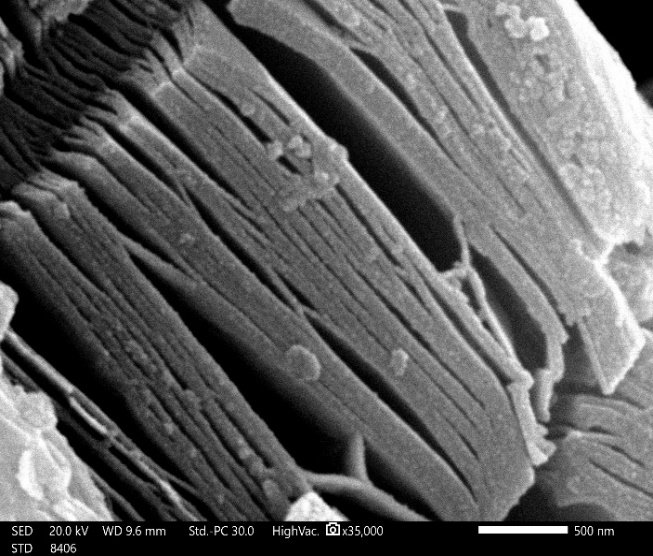  **c** | 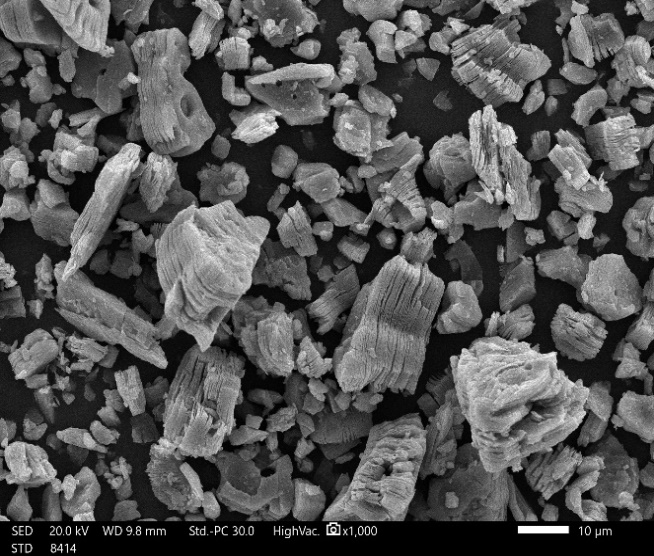  **D** |
| 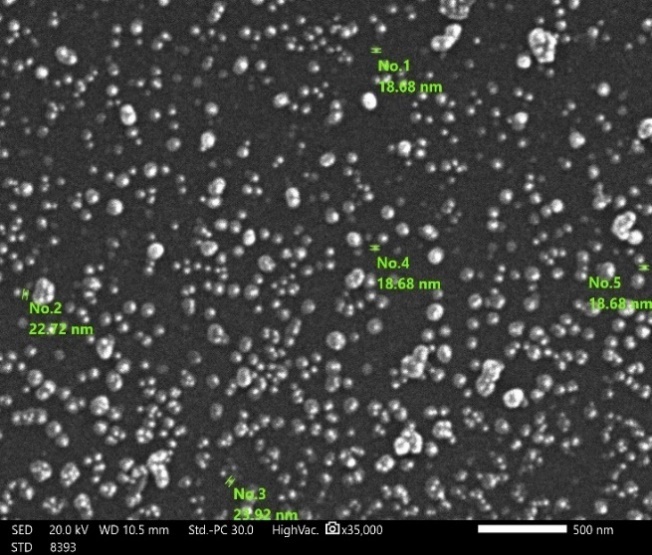  **E** | |

**Figure S5**. SEM images of (a, b) the precursor MAX phase and (c-e) 2D Ti_3_C_2_T_x_ MXene catalyst.

| 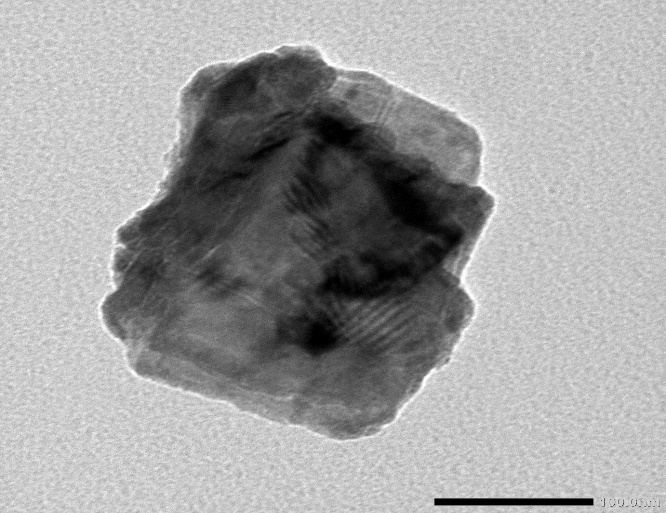  100 nm  **a** | 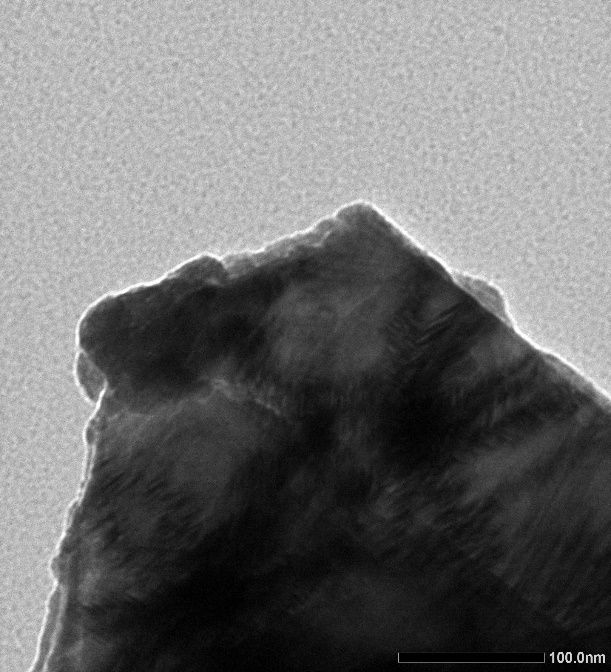  **B** |
| --- | --- |
| 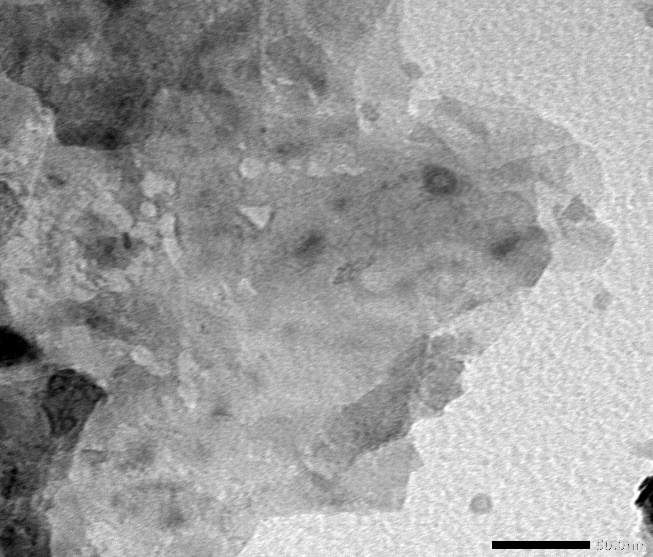  50 nm  **C** | |

**Figure S6**. TEM images of (a, b) the precursor MAX phase and (c) Ti_3_C_2_T_x_ Mxene catalyst.

**References**

1. Chen, L., et al., Measurement and analysis of thermal conductivity of Ti_3_C_2_Tx MXene films. Materials, 2018. **11**(9): p. 1701.
2. Wang, X., et al., Structure and electromagnetic properties of Ti_3_C_2_Tx MXene derived from Ti_3_AlC_2_ with different microstructures. Ceramics International, 2021. **47**(10): p. 13628-13634.
3. Garg, R., A. Agarwal, and M. Agarwal, Synthesis and optimisation of MXene for supercapacitor application. Journal of Materials Science: Materials in Electronics, 2020. **31**: p. 18614-18626.
4. Albukhari, S.M., M. Abdel Salam, and A.M. Aldawsari, Removal of malachite green dye from water using MXene (Ti_3_C_2_) Nanosheets. Sustainability, 2022. **14**(10): p. 5996.
5. Saafie, N., et al., Optimization of Synthesizing Conditions for MXene (Ti_3_C_2_) Photocatalyst: Effect of LiF: Ti_3_AlC_2_ Mass Ratio. Journal of Advanced Research in Applied Sciences and Engineering Technology, 2024. **47**(2): p. 183-192.
6. He, J., et al., Dynamically-generated TiO_2_ active site on MXene Ti_3_C_2_: Boosting reactive desulfurization. Chemical Engineering Journal, 2021. **416**: p. 129022.
7. Qu, J., et al., Preparation and regulation of two-dimensional Ti_3_C_2_T_x_ MXene for enhanced adsorption–photocatalytic degradation of organic dyes in wastewater. Ceramics International, 2022. **48**(10): p. 14451-14459.
8. Li, J., et al., Facile preparation of highly active CO_2_ reduction (001) TiO_2_/Ti_3_C_2_T_x_ photocatalyst from Ti_3_AlC_2_ with less fluorine. Catalysts, 2022. **12**(7): p. 785.
9. Zhang, X., W. Zhang, and H. Zhao, Ultrasound-assisted fabrication of Ti_3_C_2_T_x_ MXene toward enhanced energy storage performance. Ultrasonics Sonochemistry, 2022. **86**: p. 106024.
10. Dixit, P. and T. Maiti, A facile pot synthesis of (Ti_3_AlC_2_) MAX phase and its derived MXene (Ti_3_C_2_T_x_). Ceramics International, 2022. **48**(24): p. 36156-36165.
11. Rafiq, S., et al., Room-temperature synthesis of 2D-Ti_3_C_2_T_x_ nano-sheets by organic base treatment. The Journal of Chemical Physics, 2023. **158**(21).
12. Ayub, A., et al., Hydrothermal synthesis of cobalt ferrite-functionalized Ti_3_C_2_T_x_ MXene for the degradation of Congo red via peroxymonosulfate activation system. Journal of Alloys and Compounds, 2023. **963**: p. 171294.
13. Wang, Z., et al., Flexible and lightweight Ti_3_C_2_T_x_ MXene/Fe_3_O_4_@ PANI composite films for high-performance electromagnetic interference shielding. Ceramics International, 2021. **47**(4): p. 5747-5757.
14. Peng, C., et al., A hydrothermal etching route to synthesis of 2D MXene (Ti_3_C_2_, Nb_2_C): Enhanced exfoliation and improved adsorption performance. Ceramics International, 2018. **44**(15): p. 18886-18893.
